# Estimating the health and macroeconomic burdens of tuberculosis in India, 2021–2040: A fully integrated modelling study

**DOI:** 10.1371/journal.pmed.1004491

**Published:** 2024-12-12

**Authors:** Marcus R. Keogh-Brown, Tom Sumner, Sedona Sweeney, Anna Vassall, Henning Tarp Jensen

**Affiliations:** 1 Department of Global Health and Development, Faculty of Public Health and Policy, London School of Hygiene & Tropical Medicine, London, United Kingdom; 2 TB Modelling Group, TB Centre, Centre for Mathematical Modelling of Infectious Diseases, Department of Infectious Disease Epidemiology, London School of Hygiene & Tropical Medicine, London, United Kingdom; 3 Global Health Economics Centre at LSHTM, Department of Global Health, London School of Hygiene & Tropical Medicine, London, United Kingdom; 4 Department of Food and Resource Economics, Faculty of Science, University of Copenhagen, Copenhagen, Denmark; PLOS Medicine Editorial Board, UNITED STATES OF AMERICA

## Abstract

**Background:**

Tuberculosis (TB) imposes a substantial health and economic burden on many populations and countries, but lack of funding has significantly contributed to several countries falling short of global TB reduction targets. Furthermore, existing assessments of the economic impact of TB do not capture the impacts on productivity and economic growth or the pathways by which epidemiology, demography, and the economy interact. Evidence is needed to answer how investment in treatment and control measures may help to mitigate the twin Indian health and macroeconomic burdens of TB over the coming decades.

**Methods and findings:**

We develop a fully integrated dynamic macroeconomic-health-demographic simulation model for India, the country with the largest national TB burden, and use it to estimate the macroeconomic return to investment in TB treatment.

Our estimated results indicate that, over 2021 to 2040, the health and macroeconomic burdens of TB in India will include over 62.4m incident cases, 8.1m TB-related deaths and a cumulative gross domestic product (GDP) loss of US$146.4bn. Low-income households will bear larger health and relative economic burdens while larger absolute economic burdens will fall on high-income households. Achieving the World Health Organisation’s End TB target of 90% case detection could reduce clinical and demographic disease burdens by 75% to 89% and reduce the macroeconomic burden by US$120.2bn. Developing a 95% effective pan-TB treatment regimen would reduce the same burdens by 25% to 31% and US$35.3bn, respectively, while less effective but immediately achievable scaling-up of existing treatment regimens would reduce burdens by 20% to 25% and US$28.4bn, respectively. If an increase in case detection to 90% could be combined with 95% effective pan-TB treatment, it could reduce clinical and demographic disease burdens by 78% to 91% and reduce the macroeconomic burden by US$124.2bn.

In order to develop this complex integrated model framework, some aspects of the epidemiological model were simplified such that the model does not capture, for example, separate modelling of drug susceptible and multidrug-resistant (MDR) cases or separate public/private healthcare provision. However, future iterations of the model could address these limitations.

**Conclusions:**

In this study, we find that even our least effective, but most accessible, revised TB treatment regimen has the potential to generate US$28bn in GDP gains. Clearly, the economic gains of increasing case detection rates and implementing improved TB treatment regimens hinges on both the feasibility and timeframe over which they can be achieved in practice. Nevertheless, the revised TB treatment regimen is readily accessible, and our results therefore demonstrate that there is room for undertaking substantial additional investment in control and treatment of TB in India, in order to reduce the suffering of TB patients while maintaining acceptable provision of resourcing elsewhere in the Indian economy.

## Abstract 2

Marcus R. Keogh-Brown, Tom Sumner and colleagues estimate the health and macroeconomic burdens of tuberculosis in India between 2021 and 2040 in a fully-integrated dynamic macroeconomic-health-demographic simulation modelling study.

## Author summary

**Why was this study done?:**

- The health and macroeconomic burdens of tuberculosis (TB) is substantial in many countries.
- Current funding for TB is insufficient and several countries, including India, are falling short of global TB reduction targets. Existing economic tools for TB burden assessment cannot accurately guide future investment in TB control and treatment as they do not capture the interactions between epidemiology, demography, and the economy.
- Tools have been developed in other disease contexts which capture health–demographic–economic interactions to guide investment policy and, if applied to the context of TB, they could answer how investment in treatment and control measures may help to mitigate the twin Indian health and macroeconomic burdens of TB over the coming decades.

**What did the researchers do and find?:**

- We developed a model which simultaneously captures the macroeconomic, health, and demographic impacts of TB in India, the country with the largest national TB burden, and applied our model to estimate the macroeconomic return to investment in TB treatment.
- We estimated that, from 2021 to 2040, the health and macroeconomic burdens of TB in India will include over 62.4m incident cases, 8.1m TB-related deaths and a cumulative GDP loss of US$146.4bn. Our model predicts that low-income households would bear larger health and relative economic burdens while larger absolute economic burdens would fall on high-income households.
- We also found that improving case detection to 90% could reduce clinical and demographic disease burdens by 75% to 89% while developing a 95% effective pan-TB treatment regimen could reduce the same burdens by 25% to 31% and immediately scaling-up existing treatment regimens to currently achievable levels could reduce burdens by 20% to 25%.

**What do these findings mean?:**

- In spite of decade-long pursuits of strategies to end TB, the health and economic disease burdens of TB remain very high in India.
- Even our least effective, but most rapidly achievable revised TB treatment regimen has the potential to generate US$28bn in GDP gains, and thereby allow for strongly increased investment in TB control in India, which could reduce the suffering of TB patients while maintaining acceptable provision of resourcing elsewhere in the Indian economy. The potential gains of increasing case detection rates and implementing improved TB treatment regimens are substantial, but they hinge on both the feasibility and timeframe over which they can be achieved in practice.
- When developing this complex integrated model, it was necessary to simplify or limit the epidemiological aspect. We do not, therefore model drug susceptible and multidrug resistant (MDR) cases separately, neither do we separate public/private healthcare provision. However, these limitations could be addressed in future.

## Introduction

Tuberculosis (TB) imposes a substantial health and economic burden on many populations and countries [1,2]. Sadly, global TB targets remain off-track in several countries [3]. To meet these targets substantial additional funding is required, and this can be informed by estimating the full macroeconomic costs and consequences of failing to meet targets [2]. Estimating the impact of infectious diseases is challenging and requires methods that integrate macroeconomic models and infectious disease models [4]. We present an integrated epidemiological, macroeconomic, and demographic model to estimate both the health and economic burden from TB in India. India has the largest national tuberculosis burden worldwide [5], and, due to the continuing scale of the national Indian TB epidemic [3], this type of model framework is useful, both for assessing the future health and economic TB disease burdens in India, and for analysing how investment in treatment and control measures may help to mitigate these twin burdens over the coming decades.

TB is the 12th leading cause of the Global Burden of Disease and was responsible for more than 2% of that burden in 2019 [1]. The global burden of disease of TB has been reduced by more than 40% since 1990 [1], but a barrier to further reduction has been the pressure to maintain rather than increase levels of financing of TB in settings that are highly resource constrained. United Nations (UN) Sustainable Development Goals call for a 90% reduction in TB deaths by 2030, compared with 2015 [6] but meeting this target is unlikely [7]. Moreover, despite considerable increases since 2000, funding for TB is still far short of global financing targets [8] and substantial investment in research and development funding is needed for new tools to prevent TB disease among the approximately 1.7 billion people infected [7].

To achieve these targets, substantial additional investment in (a) improvements in case detection; and (b) improvements in treatment which are effective in both drug-susceptible (DS) and drug-resistant (DR) TB are needed. There are numerous microeconomic studies of TB which examine whether TB services and technologies are cost-effective from a health sector perspective, some of which include costs to both patients and households. In addition, there have been some attempts to estimate the societal value of investment in TB, which employ either preference-based value of statistical life (VSL) methods [9], or cost of illness (COI) methods [8] which estimate and scale individual costs (often including non-employed time losses) to national level indicators. Neither of these methods assess the macroeconomic impact of TB disease on productivity and economic growth (and consequently government revenues), and many do not focus on the pecuniary returns to investment in TB treatment. Moreover, previous attempts to estimate the macroeconomic impact of TB have used relatively crude methods [10,11].

We present an analysis of the health and economic burdens of TB and potential impact of investment in development and implementation of TB treatment in India. Our empirical approach fully integrates a dynamic susceptible–exposed–infectious–recovered (SEIR)-type compartmental disease model [12–15] with the type of macroeconomic computable general equilibrium (CGE) model commonly used by financing institutions and Ministries of Finance to plan investments [16]. Our approach can also be used to better understand feedback effects between health and the economy such as the much-hypothesised bidirectional relationship between TB and poverty/low-income status. We apply this integrated modelling approach to TB control in India.

## Methods

This study is reported as per the Consolidated Health Economic Evaluation Reporting Standards (CHEERS) checklist (S1 CHEERS Checklist). The model framework used in this analysis has been previously documented in [17] and consists of 3 modelling components, including a macroeconomic CGE model of the Indian economy, a set of household-specific epidemiological SEIR-type compartmental disease models of TB transmission, and a set of household-specific demographic models of the Indian population. These models are outlined briefly in the following subsections and are fully integrated within 1 model framework such that feedback effects between the sub-models can be captured endogenously. Further details on the structure and parameters can be found in S1 Appendix.

### Macroeconomic modelling component

We employ a multi-sector and recursively dynamic macroeconomic CGE model of the Indian economy. CGE models arrive at their outputs by capturing the behaviour of different agents in the economy, of which there are 4 main economic agents. Firms seek to combine resource inputs to maximise profits, consumers allocate their disposable income between consumption and savings to maximise welfare, the government levies taxes, distributes benefits and purchases goods directly and foreign agents interact with domestic agents through goods trade (imports/exports), international factor income flows, foreign unrequited transfers and foreign borrowing and lending. As such, the model provides a characterisation of the whole economy and the interactions and trade-offs within it.

Our macroeconomic CGE model was developed from the International Food Policy Research Institute (IFPRI) Standard Model framework [18] and calibrated to a 2014 Social Accounting Matrix (SAM) for India. The SAM was derived from the Global Trade Analysis Project’s GTAP10 database [19]. The static model was made recursively dynamic via the introduction of capital and labour factor updating equations. The capital updating equations were calibrated to capital stock and depreciation data from the Penn World Tables 10.0 [19,20] while the labour updating equations are linked to the demographic model [17]. The macroeconomic model distinguishes 3 factors of production, including 2 labour factors (skilled and unskilled labour) and capital. In addition, the 68th National Sample Survey 2011–12 for India [21] was used to disaggregate the households in the model into 5 representative households, stratified according to income quintiles. Once the dynamically recursive model was fully specified, it was run forward to 2020 by targeting 2015 to 2020 nominal and real gross domestic product (GDP) aggregates from the World Development Indicators database [22] and to establish 2021 as our base year for the 2021 to 2040 simulations.

### Demographic modelling component

Similar to the CGE sub-model, the demographic sub-model includes 5 representative households, and, similar to the CGE model, the 5 households are derived from the 68th National Sample Survey 2011–12 for India [21]. The household-specific demographic sub-models are calibrated to the Indian population projections from the World Population Prospects, 2019 Revision [23], and each sub-model include specifications for calculating fertility (births), mortality, net immigration, and population levels, and spans 17 five-year age categories and 2 gender types.

### TB modelling component

The TB model framework includes specifications for the transition of the population between different health states and provides estimates of TB cases and deaths as inputs to the macroeconomic and demographic models. To facilitate linkage with the other modelling components, the TB model is kept as simple as possible, while representing the key aspects of TB epidemiology and control in India and incorporating key health pathways between the economic and epidemiological models. A brief description of the TB model is given below, while more details can be found in S1 Appendix and in [17].

The structure of the core SEIR model is similar to many previously published TB models [12–15,24]. Susceptible individuals can be infected and either develop primary disease or enter a latently infected state. Latently infected individuals may subsequently progress to disease or be re-infected. Those with prevalent disease may be diagnosed and start treatment, spontaneously recover or die due to TB. Those started on treatment may be successfully treated and return to the latent state, fail treatment, or be lost to follow-up, and return to the disease state, or die while on treatment. Those who fail treatment are returned to a separate compartment (in order to avoid double-counting) from where they can either be re-treated or die. Details of the compartment-structure and all parameter values are given in S1 Appendix.

Similar to the other modelling components, the TB model is stratified into 5 household types by wealth quintiles, based on data from the Indian National Family Health Survey (NFHS)-4 2015–16 [25], in order to capture differences in TB burden by socioeconomic status (SES). The risks of developing TB differ by SES (due to differences in the prevalence of risk factors) and we also account for differences in contact rates within and between household types (see S1 Appendix).

Specifically, we use data on the prevalence of 6 risk factors (smoking, exposure to indoor air pollution, low body mass index (BMI), alcohol use, diabetes, HIV) from the NFHS-4 [25], and estimates of the relative risk of TB associated with these risk factors [26], to establish the baseline risk factor parameters and exposures by household type. In contrast to the remaining risk factors which are kept exogenous, the household-specific “low BMI” risk factors are endogenously determined by changes in household-specific nutritional food demand intakes in the macroeconomic model, thereby providing a feedback loop and ensuring full integration between all model components [17]. The TB model is fitted using a root-mean-squared-deviations metric to match central World Health Organisation (WHO) estimates of TB incidence, deaths due to TB, overall TB prevalence, and TB prevalence by SES in 2021 [27], by varying the rate of diagnosis and the within and between household contact parameters. Multidrug-resistant (MDR) TB cases are assumed to account for a constant 3% share of notified TB cases, based on national notification and treatment data reported to WHO [28].

We conservatively assume that each drug susceptible individual would be unable to work for an average of 8 weeks during the intensive and continuation phases of treatment and is assumed to receive treatment at a full treatment cost of US$136.15 (2018 US$) [29]. Those individuals with MDR TB are assumed to be unable to work for an average of 9 weeks and receive treatment at a full treatment cost of US$1,229.29 (2018 US$) [29]. Estimates of work time loss, which are based on changes in employment rates, approximate absenteeism from work but do not account for additional losses due to presenteeism and reduced productivity at work or work time loss post-TB treatment. Health service costs are adjusted to 2021 prices (the base year of the model). For further details on cost data sources and work absence, see S2 Appendix.

We present economic results in both real terms (2021 prices), and in net present value (NPV) terms using a real discount rate of 5.7%, which mirrors the average real interest rate over 2013–20 in India [22]. Details of our macroeconomic closures and detail on our model calibration and specification are given in a previous application of this model framework [17].

### Modelling scenarios

We use our integrated model framework to simulate the pecuniary health-related macroeconomic impact of each of the scenarios described below. Our focus on pecuniary costs means that we include all health costs, but only value productivity losses by employed individuals. This approach provides an important key to intepretation of our results since it allows for our macroeconomic impacts to be interpreted as the total economic return available to be reaped from development and implementation of treatment and control measures, if the planned policy-related reductions in TB were acomplished in reality.

### Scenario: Disease burden (elimination of TB)

Our “disease burden” scenario consists of simulating the removal of the burden of TB on the Indian economy over the period 2021 to 2040. Practically, this is accomplished by first establishing a counterfactual growth path, and subsequently removing the associated costs to the healthcare sector for all incident cases together with the effects of morbidity and mortality-related labour supply losses on workers. This allows our model to estimate the impact of an ideal scenario of TB elimination for comparison with the impacts of our treatment scenarios (see below) and, thereby, to establish the scope for investment in development and roll-out of these policy programmes to reduce the TB burden in India.

### Scenario: Improved treatment (pan-TB)

In our “improved treatment” scenario, we simulate the effect of the immediate introduction of a new pan-TB regimen for treating all forms of TB. Based on the WHO target regimen profile (TRP) [30,31], and consistent with previous modelling of deployment of a pan-TB drug regimen in India, we assume the introduction of the regimen would result in an increase in treatment success rate to 95% for all forms of TB (compared with a value of 80% in our counterfactual simulation). While we do not explicitly model the details of such a regimen, the WHO TRP [31] proposes that a pan-TB regimen should: consist of a maximum of 4 months of once daily oral medications; require no active monitoring for drug toxicity; have no drug–drug interactions with antiretroviral regimens; not contain rifampicin, isoniazid, or pyrazinamide (so require no testing for rifampicin resistance prior to treatment initiation).

### Scenario: Improved diagnosis (case detection)

We also simulate the effects of immediate improvements in case detection rates. Current estimates suggest that approximately 63% of incident TB cases in India are diagnosed and started on treatment [32]. We modelled an increase in this proportion to 90%, the minimum target defined in the WHO End TB Strategy [33]. This is implemented in the model as an increase in the rate of diagnosis (see S1 Appendix).

### Scenario: NTEPI target for improvements in existing treatment implementation (Scaling Existing Treatment)

The pan-TB intervention modelled above is based on an optimistic treatment improvement. There is also debate about the potential adverse consequences of a pan-TB regimen. The National TB Elimination Programme in India’s (NTEPI) National Strategic Plan for Tuberculosis Elimination 2017–2025 provides targets for treatment success rates which could be achieved through improvements in the delivery of existing treatment regimens including access to free TB drugs for all patients, improved patient friendly adherence monitoring, and nutritional and financial support for patients taking TB treatment [34]. The revised treatment outcome targets are 92% for DS-TB and 75% for DR-TB by 2025, which, adjusting for our assumption that 3% of TB cases are DR, yields an overall average of 91.5%. We therefore model this reduced efficacy scenario as an additional treatment scenario.

### Sensitivity analysis

In order to test the robustness of our results to variations in key parameters, we include sensitivity analyses of our disease burden and treatment scenarios with respect to recalibrations of our household-specific TB models to target variations in TB morbidity and mortality. The WHO estimated confidence bands around the central TB morbidity and mortality estimates [27] which we used to calibrate our baseline model. Specifically, the WHO estimated that Indian TB incidence in 2021 was 200 per 100,000 with a confidence interval of [172; 230] per 100,000; and that Indian TB mortality was 26 per 100,000 with a confidence interval of [19; 34] per 100,000 [27]. Apart from the combination of central estimates, which we used to calibrate our baseline TB models, we re-calibrated our integrated model framework to target all of the other 8 permutations of lower/central/upper bounds of 2021 Indian TB incidence and TB mortality, and re-simulated each of the policy scenarios, outlined above, for each of the re-calibrations, to provide a robust test of the sensitivity of our results.

Since it has been suggested that TB patients may be less productive at work before treatment, we also conduct sensitivity analysis to allow for pretreatment presenteeism of TB patients. Previous estimates suggest that, prior to treatment, TB patients are less productive and this productivity loss could on average amount to 48 pretreatment workdays [35]. We therefore re-simulated our central disease burden and policy scenario results including the additional assumption that every TB patient, who would have otherwise worked, would be less productive, and that their loss of productivity would be equivalent to a loss of 48 pretreatment working days.

## Results

### Disease burden scenario results

**Fig 1:**
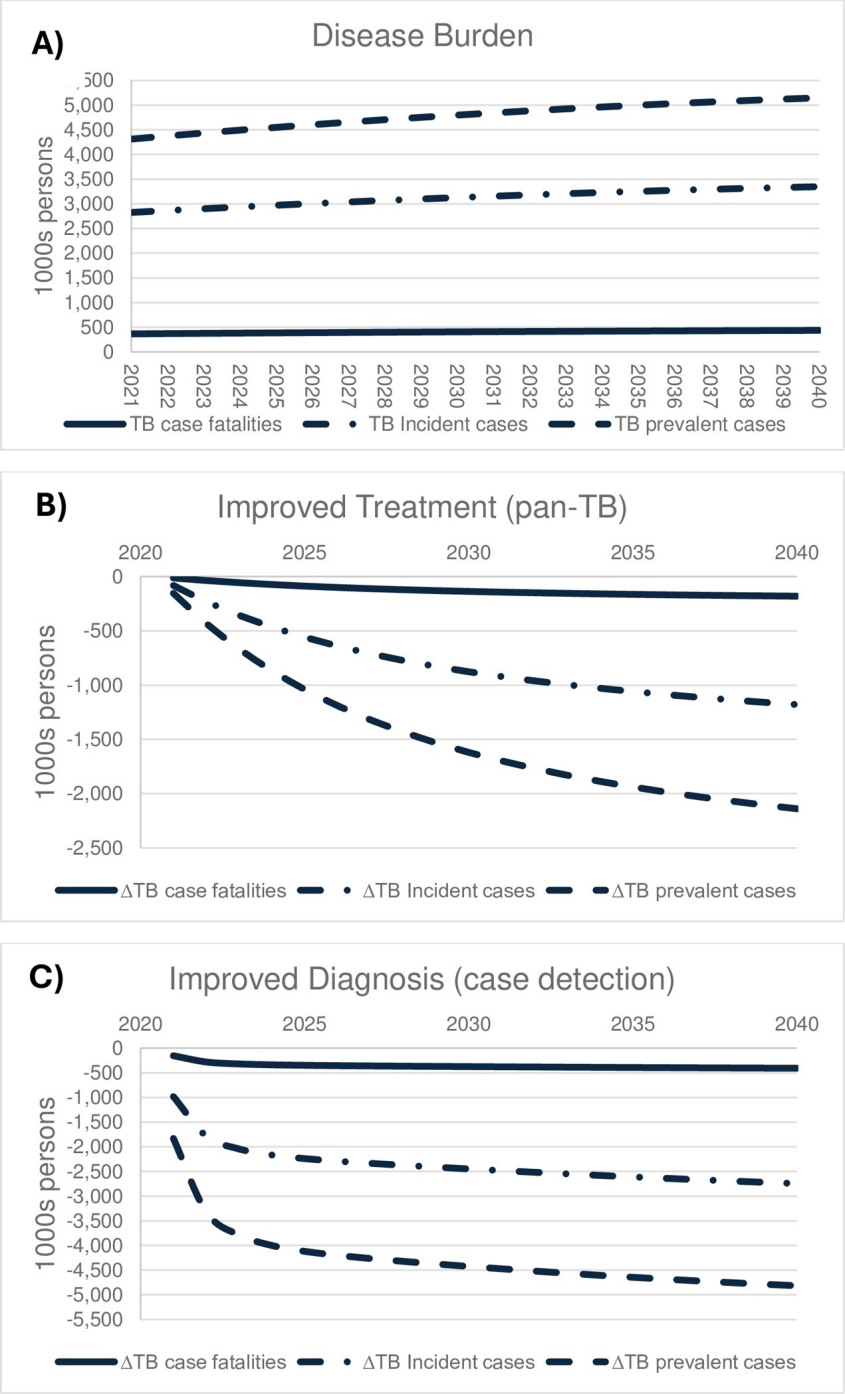
Estimated clinical health outcomes from TB: (A) Disease burden, (B) improved treatment (pan-TB), (C) improved diagnosis (case detection).

Our counterfactual clinical health outcomes from TB over the period 2021 to 2040 are presented in Fig 1A and the cumulative outcomes estimated by the TB modelling component are presented in the clinical, epidemiological, and demographic half of Table 1. The cumulative values amount to an estimated population reduction of 80m person-years resulting from 8.1m case fatalities and 62.4m incident cases over our 20-year simulation period. Table 1 also shows that, for the broader India population, the projected future TB disease burden cumulatively includes 18.2bn person-years in a susceptible state, 11.7bn person-years in a latently infected state, 95.8m person-years in an infectious state, and 25.4m person-years undergoing treatment for TB; and TB would cause 7.0m excess deaths over 20 years, which is smaller than the 8.1m reduction in case fatalities, since some individuals, who avoid death via TB disease, would die from other causes.

**Table 1:** Estimated 2021–2040 economic, clinical, epidemiological, and demographic impacts ‐ pan-TB treatment and 90% case detection scenarios (Δ = change relative to business as usual, #person-years = number of person years, NPV = net present value, avg = average).

	Disease Burden (DB*)		Scenario: pan-TB treatment			Scenario: 90% case detection			Scenario: combined		
Indicators											
**ECONOMIC**											
GDP PER CAPITA (USD)	USD	% change	USD	% change	% of DB*	USD	% change	% of DB*	USD	% change	% of DB*
- ΔReal GDP/capita/year	−4.21	−0.217%	1.02	0.053%	−24.3%	3.47	0.179%	−82.4%	3.59	0.185%	−85.1%
NPV of GDP (2021–2040) (bn USD)	bn USD	% change	bn USD	% change	% of DB*	bn USD	% change	% of DB*	bn USD	% change	% of DB*
- ΔNPV GDP	−146.4	−0.215%	35.3	0.052%	−24.1%	120.2	0.176%	−82.1%	124.2	0.182%	−84.8%
- ΔNPV GDP (Treatment Costs)	−15.7	−0.023%	3.2	0.005%	−20.6%	11.2	0.016%	−71.0%	11.5	0.017%	−73.4%
- ΔNPV GDP (ΔLabour supply ‐ Morbidity)	−18.4	−0.027%	4.4	0.006%	−23.9%	13.4	0.020%	−72.9%	13.8	0.020%	−74.8%
- ΔNPV GDP (ΔLabour supply ‐ Mortality)	−113.3	−0.166%	27.6	0.040%	−24.3%	95.6	0.140%	−84.3%	98.8	0.145%	−87.2%
NPV of household income and consumption (2021–2040) (bn USD)	bn USD	% change	bn USD	% change	% of DB*	bn USD	% change	% of DB*	bn USD	% change	% of DB*
- ΔNPV Household income	−116.8	−0.171%	28.1	0.049%	−24.1%	95.9	0.166%	−82.1%	99.1	0.172%	−84.8%
- ΔNPV Labour income	−66.7	−0.098%	15.9	0.042%	−23.8%	54.6	0.144%	−81.9%	56.5	0.149%	−84.6%
- ΔNPV Capital income	−50.1	−0.073%	12.2	0.062%	−24.4%	41.3	0.208%	−82.4%	42.6	0.215%	−85.1%
- ΔNPV Household consumption	−102.2	−0.150%	24.7	0.057%	−24.1%	83.9	0.194%	−82.1%	86.7	0.200%	−84.8%
Labour market (1,000s person-years)	1,000s	% change	1,000s	% change	% of DB*	1,000s	% change	% of DB*	1,000s	% change	% of DB*
- ΔUnskilled labour (#person-years)	−28,627.7	−0.313%	7,178	0.079%	−25.1%	24,026	0.263%	−83.9%	24,796	0.271%	−86.6%
- ΔSkilled labour (#person-years)	−4,496.3	−0.222%	1,114	0.055%	−24.8%	3,756	0.186%	−83.5%	3,879	0.192%	−86.3%
**CLINICAL, EPIDEMIOLOGICAL and DEMOGRAPHIC**											
Demographic outcomes	1000s	% of total	1000s	% of total	% of DB*	1000s	% of total	% of DB*	1,000s	% of total	% of DB*
- ΔPopulation (1,000s prs-years)	−80,029.5	−0.27%	19,891	0.07%	−24.9%	68,114	0.23%	−85.1%	70,376	0.23%	−87.9%
- Excess deaths (1,000s persons)	7,042.4	3.58%	−2,182	−1.11%	−31.0%	−6,274	−3.19%	−89.1%	−6,425	−3.27%	−91.2%
Clinical outcomes (1,000 persons)	1,000s	% change	1,000s	% change	% of DB*	1,000s	% change	% of DB*	1,000s	% change	% of DB*
- ΔTB incident cases	62,424	-	−16,158	−25.9%	−25.9%	−47,272	−75.7%	−75.7%	−48,455	−77.6%	−77.6%
- ΔTB case fatalities	8,102	-	−2,512	−31.0%	−31.0%	−7,208	−89.0%	−89.0%	−7,383	−91.1%	−91.1%
Cumulative population by compartment (mm pers-yrs†)	mm	% change	mm	% change	% of DB*	mm	% change	% of DB*	mm	% change	% of DB*
- ΔSusceptible population	18,239.3	-	554.2	3.0%	3.0%	1,955.2	10.7%	10.7%	2,021.9	11.1%	11.1%
- ΔLatently infected population	11,703.2	-	−497.0	−4.2%	−4.2%	−1,787.6	−15.3%	−15.3%	−1,847.9	−15.8%	−15.8%
- ΔInfectious population ‡	95.8	-	−29.7	−31.0%	−31.0%	−85.2	−89.0%	−89.0%	−87.3	−91.1%	−91.1%
- ΔTreatment population $	25.4	-	−7.6	−30.0%	−30.0%	−14.2	−56.1%	−56.1%	−16.3	−64.2%	−64.2%
Risk factors (endogenous)	%-points	% change	%-points	% change	% of DB*	%-points	% change	% of DB*	%-points	% change	% of DB*
- Δlow BMI prevalence (avg)	20.45%	-	−0.31%	−1.5%	−1.5%	−1.04%	−5.1%	−5.1%	−1.08%	−5.3%	−5.3%
Memorandum Items (2021–2040):											
- Real GDP/capita/year (USD)	1,939										
- NPV GDP (bn USD)	68,198										

Notes: Own calculations.

*We use “DB” to refer to “Total Disease Burden impact” on any given economic, epidemiological, and demographic indicator.

†We use “mm prs-yrs” to refer to millions of cumulative person-years over our 2021–40 time horizon.

‡The infectious population consists of both newly infectious and previously unsuccessfully treated persons.

^$^The treatment population consists of both persons who are newly treated and re-treated after prior unsuccessful treatment.

Macroeconomic impacts estimated by our integrated model framework indicate that the economic TB disease burden over our 2021 to 2040 simulation period (Table 1) would amount to an annual real GDP per capita loss of US$4.21 (0.22%) and cumulative 2021–40 NPV GDP loss of US$146.4bn. The main determinant of the predicted macroeconomic NPV GDP disease burden is mortality-related labour supply losses, which constitutes approximately 77% (US$113.3bn) of the burden. This is because morbidity impacts are temporary, lasting a fraction of the year, whereas mortality-related losses are cumulative, lasting for the number of years that a working age individual could, otherwise, have worked from the time of their death until retirement age (or until the end of the simulation). The remainder of the predicted NPV GDP burden is made up approximately equally from NPV GDP impacts of morbidity-related labour supply losses (12.5%, US$18.4bn) and NPV GDP impacts of TB treatment costs (12.6%, US$15.7bn).

The predicted burden on Indian household incomes amounts to US$116.8bn (Table 1), and the breakdown across household income quintiles (Table 2) indicates that the burden is progressive in absolute terms (highest absolute burden falling on the highest income quintile Q5; US$69.0bn) and regressive in relative terms (highest relative burden falling on the lowest income quintile Q1; 0.45%). For non-economic indicators, the TB burden is regressive, in both absolute and relative terms, resulting from greater exposure to key TB risk factors by low-income households, particularly smoking, low BMI, and indoor air pollution (see Table E in S1 Appendix). For example, simulated population and labour force impacts (Tables 1 and 2) shows that the total population burden (80m person-years) would fall disproportionately on Q1 (22.0m person-years) compared with Q5 (10.4m person-years), and similarly that the total labour force burden (33.1m work-years) would fall disproportionately on Q1 (8.9m work-years) compared to Q5 (4.4m work-years).

**Table 2:** Estimated 2021–2040 cumulative household impacts ‐ TB Disease Burden (bn USD; 2021 prices) (Δ = change relative to business as usual, #person-years = number of person years, NPV = net present value).

	Households									
	Income Q1		Income Q2		Income Q3		Income Q4		Income Q5	
ECONOMIC DISEASE BURDEN	bn USD	% of base	bn USD	% of base	bn USD	% of base	bn USD	% of base	bn USD	% of base
- ΔNPV Household income	9.3	0.45%	10.0	0.29%	11.5	0.22%	17.1	0.18%	69.0	0.19%
- ΔNPV Labour income	9.0	0.46%	9.1	0.30%	9.4	0.21%	11.6	0.15%	27.7	0.13%
- ΔNPV Capital income	0.3	0.25%	0.9	0.25%	2.2	0.25%	5.5	0.25%	41.3	0.25%
- ΔNPV Household consumption	9.0	0.48%	9.9	0.32%	11.5	0.25%	17.0	0.21%	54.9	0.22%
LABOUR MARKET	1,000s	% of base	1,000s	% of base	1,000s	% of base	1,000s	% of base	1,000s	% of base
- ΔUnskilled labour (#person-years)	8,505	0.55%	7,409	0.38%	5,901	0.29%	4,171	0.21%	2,642	0.17%
- ΔSkilled labour (#person-years)	404	0.55%	621	0.38%	714	0.29%	972	0.21%	1,786	0.17%
DEMOGRAPHY	1,000s	% of base	1,000s	% of base	1,000s	% of base	1,000s	% of base	1,000s	% of base
- ΔPopulation (#person-years)	22,040	0.47%	19,377	0.34%	15,911	0.26%	12,286	0.19%	10,416	0.15%
- Excess deaths (#persons)	−1,857	−5.02%	−1,752	−5.59%	−1,428	−4.06%	−1,095	−2.69%	−910	−1.74%
TB CLINICAL OUTCOMES	1,000s	% of total	1,000s	% of total	1,000s	% of total	1,000s	% of total	1,000s	% of total
- TB incident cases (#persons)	−16,591	27%	−15,411	25%	−12,586	20%	−9,706	16%	−8,130	13%
- TB case fatalities (#persons)	−2,154	27%	−2,001	25%	−1,633	20%	−1,259	16%	−1,055	13%
Note: Own calculations										

The absolute estimated economic burdens are progressive since spillover effects, in the form of reduced accumulation of capital assets, fall primarily on Q5 households, and since losses of skilled labour are particularly costly for Q5 households. Hence, the distributional impacts on household incomes are generally attributable to the interaction between ownership of capital and the quantity and skill level (or value) of labour supply losses. Table 2 demonstrates that 82.4% of total capital income losses would fall on the wealthiest Q5 households (US$41.3bn), while less than 1% would fall on the lowest income Q1 households (US$0.3bn). Predicted labour income losses are also higher for wealthier households since, despite their 50% lower overall workforce losses, up to 5 times as many high-wage skilled workers would be affected by TB in high-income Q5 households, which carries a higher total cost. TB therefore places higher health and relative income burdens on low-income households but larger total costs on high-income households.

### Improved treatment (pan-TB) scenario results

An improved pan-TB treatment scenario is expected to raise the TB treatment success rate from 80% to 95%. We find that (Table 1) across the Indian population, a pan-TB regimen would lead individuals to spend 31% less time, on average, being infectious and 30% less time being on treatment. Overall, the introduction of a pan-TB treatment would increase the Indian population by approximately 19.9m person-years, save individuals from 37.3m person-years of TB illness, and avoid 16m incident cases and reduce case fatalities by more than 2.5m during 2021–40; and excess deaths would be reduced by 2.2m. Over time, annual clinical benefits would increase (Fig 1B), implying that introducing a pan-TB treatment would address 3% to 4% of the annual clinical disease burden initially, and remove 35% to 42% of the annual clinical disease burden by 2040, amounting to cumulative removal of one quarter of the TB disease burden incident cases and 31% of case fatalities over 20 years (Table 1).

Compared with business as usual, a pan-TB treatment is expected to reduce clinical and demographic (population numbers and excess deaths) impacts. It would also expand labour supplies by 7.2m unskilled and 1.1m skilled worker-years and increase annual Indian GDP per capita by US$1.02 and cumulative 2021–40 NPV GDP by US$35.3bn (equivalent to a 24% reduction of the economic TB disease burden). It is also worth noting that the income expansion leads to a predicted expansion in household (food) consumption and nutritional intakes, and this leads to an overall reduction in the endogenous “low BMI prevalence” risk factor by 1.5% (Table 1). This positive feedback effect represents a nutritional externality, and it results from the full integration of the economic and epidemiological sub-models. It thereby demonstrates how our full model integration captures (nutritional) externalities which reinforce the clinical and demographic, and by implication economic, benefits of introducing a pan-TB treatment.

A decomposition analysis indicates that the predicted NPV GDP gains from a pan-TB treatment can be attributed, mainly, to reductions in mortality (US$27.6bn) and with smaller but still significant contributions from morbidity-related labour losses (US$4.4bn) and reduced healthcare costs (US$3.2bn). Equivalently, our analysis suggests that if a pan-TB treatment could be successfully developed and implemented in India, for less than US$35bn, it would both be beneficial for the Indian economy, and bring with it substantial additional reductions in human suffering for the Indian population.

**Fig 2:**
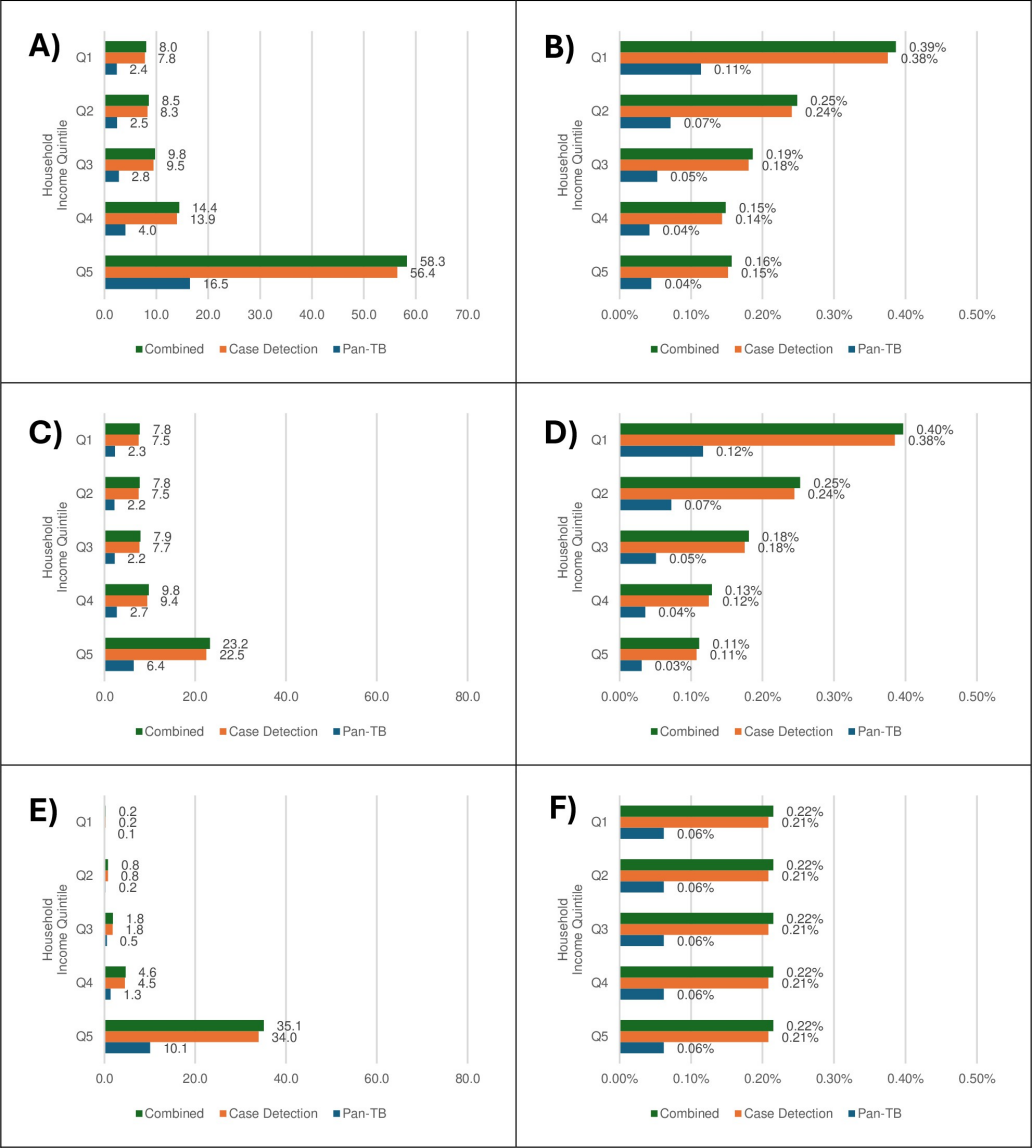
Estimated TB treatment scenario impacts: Household income composition and distribution. (A) ΔTotal Household Income (bn USD), (B) ΔTotal Household Income (% change), (C) ΔHousehold Labour Income (bn USD), (D) ΔHousehold Labour Income (% change), (E) ΔHousehold Capital Income (bn USD), (F) ΔHousehold Capital Income (% change) (Δ indicates change relative to business as usual).

**Fig 3:**
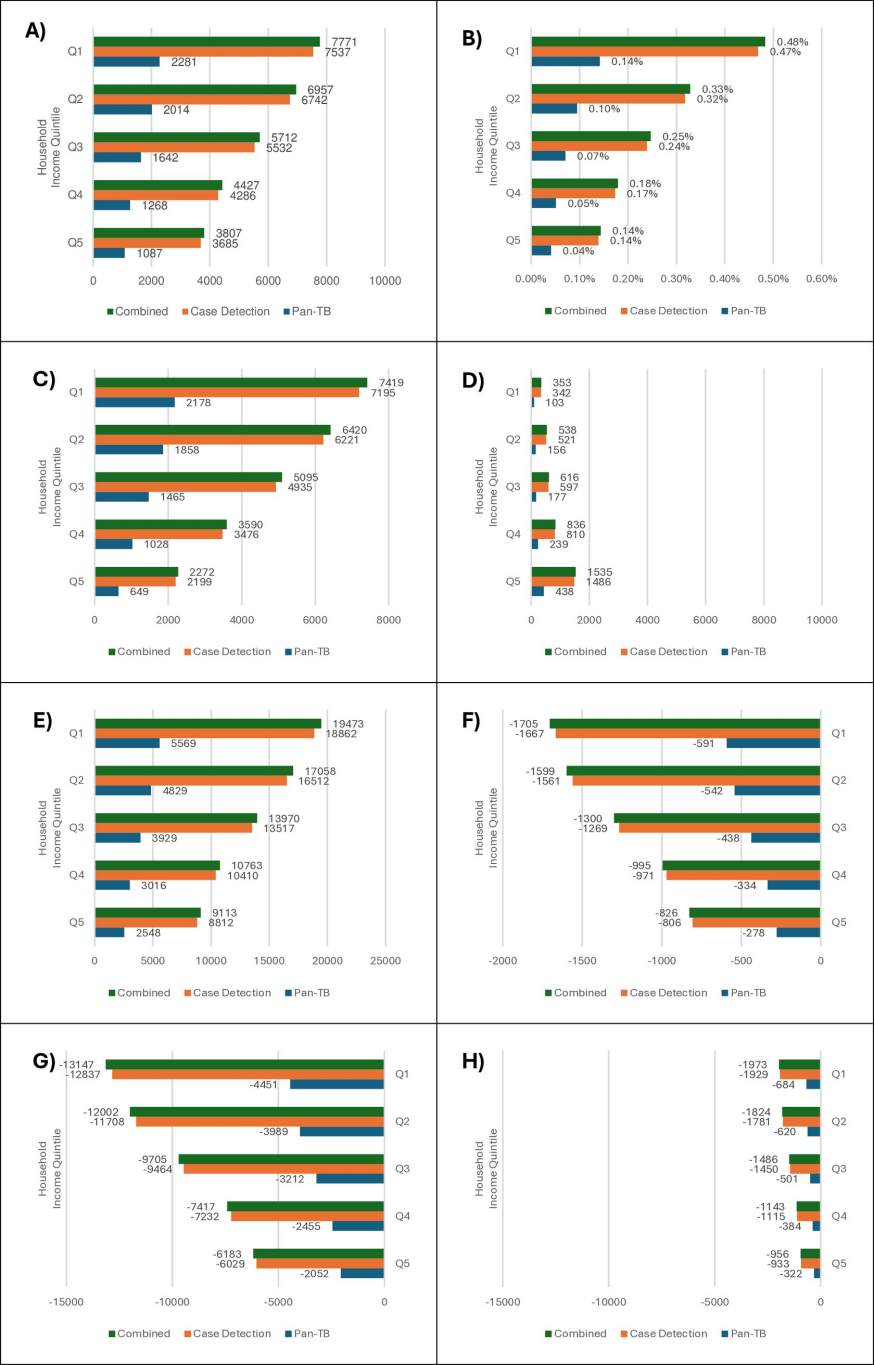
Estimated TB treatment scenario impacts–labour market, demographics, and clinical outcomes. (A) ΔTotal labour supplies (1,000s work-years), (B) ΔTotal labour supplies (% changes), (C) ΔUnskilled labour supplies (1,000s work-years), (D) ΔSkilled labour supplies (1,000s work-years), (E) ΔPopulation (1,000s person-years), (F) ΔExcess Deaths (1,000s persons), (G) ΔTB incident cases (1,000s persons), (H) ΔTB case fatalities (1,000s persons) (Δ indicates change relative to business as usual).

Due to the distribution of disease burdens across household income quintiles, it is no surprise that our simulated pan-TB treatment would benefit the wealthier Q5 households more in absolute economic terms, while it would generally benefit the poorer Q1 households more in clinical, demographic, and relative economic terms (Figs 2 and 3). In terms of clinical and demographic outcomes, there would be progression in distributional impacts for all indicators (Fig 3), including incident cases and fatalities, where reduced burdens range from respectively 2.1m cases and 322,000 fatalities in the Q5 to 4.4m cases and 684,000 fatalities in Q1 households; avoided excess deaths which range from 278,000 in Q5 to 591,000 in Q1 households; and saved life-years which range from 2.5m person-years in Q5 to 5.6m person-years in Q1 households over 20 years. The only regressive demographic indicator is skilled labour supplies (i.e., working-age population corrected for labour force participation rates) which improves more for Q5 (438,000 work-years) compared to Q1 (103,000 work-years). In economic terms, the distributions of absolute benefits are regressive (Fig 2), bringing the greatest NPV household income gains to wealthier Q5 households (US$16.5bn) and lower gains to poorer Q1 households (US$2.4bn). A decomposition analysis shows that this is driven by regressive distributions of both absolute capital and (skilled) labour income gains. In contrast, relative NPV household income gains are progressive, with wealthier Q5 households benefitting less (0.04%) compared to poorer Q1 households (0.11%).

### Improved diagnosis (case detection)

Our improved diagnosis scenario simulated improvements in diagnostic services to achieve 90% treatment coverage (the proportion of all (DS and MDR) incident TB cases diagnosed and started on treatment).

Our estimated results (Table 1) indicate that an increased diagnostic success rate would lead to massive reductions in clinical and demographic TB disease burdens. For example, across the Indian population, individuals would, on average, spend 89% less time being infectious (approximately 3 times the reduction from the introduction of a pan-TB treatment) and 56% less time undergoing treatment. Overall, if the minimum WHO target of 90% case detection is achieved, the improved case detection would save the Indian population from 99.4m person-years of TB illness and avoid more than 47m incident cases and reduce case fatalities by 7.2m during 2021–40, and it would increase the Indian population by more than 68m person-years and avoid 6.3m excess deaths.

Compared to a pan-TB treatment intervention, the clinical impacts of the simulated 90% case detection rate intervention are more substantial, and, in a dynamic perspective, the effect occurs more rapidly (Fig 1C). Hence, second year (2022) clinical outcomes of the increased case detection rate would remove 62% of the annual incidence burden and 76% of the annual fatalities and prevalent cases, and by 2040, the annual clinical outcome burdens would be reduced by 82% for the annual TB incident cases and 94% for annual fatalities and prevalent cases.

Turning to labour market outcomes, the increased case detection rate, and resulting clinical outcome improvements, are predicted to remove roughly 82% of the labour market NPV GDP disease burdens for both skilled and unskilled workers, and, together with reduced healthcare costs, increase annual Indian GDP per capita by US$3.47 and cumulative 2021–40 NPV GDP by US$120.2bn. Similar to the pan-TB treatment impacts, it is worth noting that the significant income expansion would, again, lead to an expansion in household (food) consumption and nutritional intakes, and, for the current scenario, this would lead to an overall reduction in the endogenous “low BMI prevalence” risk factor by 5.1% (Table 1). The stronger nutritional externality further reinforces the clinical and demographic gains and by extension the NPV GDP gains. A decomposition analysis indicates that the NPV GDP gains can be attributed, mainly, to averted labour force mortality (US$95.6bn), and, to a lesser extent, to morbidity (US$13.4bn) and healthcare savings (US$11.2bn).

Due to the distribution of disease burdens across household income quintiles, improved case detection is, again, expected to benefit the wealthier Q5 households more in absolute economic terms, but benefit the poorer Q1 households more in clinical, demographic, and relative economic terms (Figs 2 and 3). When compared with our estimated pan-TB treatment impacts, we can also see that the predicted household-level impacts of improved case detection are, on a larger scale, driven by greater health improvements. The heavier clinical and demographic burdens of TB on low-income Q1 households, which is attributable to their higher exposure to TB risk factors, allows for greater clinical and demographic gains including 12.8m avoided incident cases and 1.9m avoided fatalities, and labour market gains of 7.5m worker-years. Both absolute and relative non-economic burden reductions would be generally smaller for high-income Q5 households. The only exception is skilled labour supplies, where Q5 households would gain 1.5m skilled work-years while poorer Q1 households only would gain 342,000 skilled work-years. Compared to the improved treatment scenario, capital income gains again make up 60% of wealthier household gains and these combine with higher gains from labour income which are driven by skilled labour, resulting in greater overall gains for higher-income households. Lower-income households again experience larger relative income gains to accompany their greater share of the health benefits.

### Combined scenario (improved treatment and case detection)

To assess potential interaction effects, and the importance of pursuing a two-pronged strategy rather than prioritising one strategy over the other, we simulate a scenario which combines the planned introduction of an improved pan-TB treatment regimen with our improved case detection scenario. We find that the combined strategy would eliminate more of the TB burden than the individual interventions in isolation. However, our results also show strongly negative interaction effects, suggesting that improvements to case detection rates reduce the marginal effect of introducing a pan-TB treatment and vice versa. For example, the combined scenario reduces the average time, which the Indian population spends undergoing treatment, by 64%. This is significantly lower than the sum of the individual scenario impacts for a pan-TB treatment (30%) and improved case detection (56%), demonstrating that if the case detection rate is initially raised to the WHO minimum standard level, the added marginal benefit of introducing a pan-TB treatment regimen would be expected to lower average time spent on treatment by 8% (rather than 30%).

In terms of other clinical and demographic impacts, the combined intervention is predicted to save the Indian population from 104m person-years of TB illness, avoid 48.5m incident cases, and reduce case fatalities by 7.4m during 2021–40; and it would increase the Indian population by over 70 million person-years, and lower excess deaths by 6.4m, thereby removing respectively 78% and 91% of the population and excess death burdens of TB. It can, again, be noted that the simulated 90% case detection intervention would, by itself, increase the Indian population by nearly 68m person-years and reduce excess deaths by 6.3m, pointing to the conclusion that, if the target 90% case detection rate is deemed achievable, then development and implementation of a pan-TB treatment would only need to be given priority in order to improve treatment of TB illness, since the prevention impact of the 90% case detection rate target, once fully implemented, is close to being instantaneous and would therefore marginalise the longer term prevention impact of a pan-TB treatment. On the other hand, if attainment of the 90% case detection rate target is deemed unachievable, the longer-term prevention effect would be a strong additional argument for pursuing development and implementation of a pan-TB regimen in India.

Turning to the estimates of economic impacts, the combined scenario yields potential annual GDP per capita gains of US$3.59 and a cumulative NPV GDP increase of US$124.2bn over 2021 to 2040. The strong reductions in incident cases and deaths of TB, noted above, would lead to 29m gained work-years of predominantly unskilled labour, and, together with healthcare savings, this drives the economic impacts. A decomposition analysis shows that the predicted NPV GDP gain is composed of a US$98.8bn gain due to averted labour supply mortality, a US$13.8bn gain due to averted labour morbidity, and a US$11.5bn NPV GDP gain due to reduced healthcare spending. Again, strongly negative interaction effects indicate that concomitant implementation of the 2 component interventions would strongly reduce the marginal economic impact of either intervention.

The distribution of disease burdens across household income quintiles means that the combined scenario, as with our component scenarios, would benefit the wealthier Q5 households, more, in absolute economic terms, while the poorer Q1 households generally benefit more in clinical, demographic, and relative economic terms (Figs 2 and 3). Poorer Q1 households would see larger clinical health and demographic benefits due to their higher initial burden of TB, including a 19m person-year increase in population and a 7.8m work-year increase in labour supply (mostly unskilled). Apart from skilled labour supply gains, the wealthiest Q5 households’ health and demographic gains are generally half those of the poorest Q1 household group. Estimates of absolute economic impacts are also, as for the component interventions, regressive for the combined scenario. Total income gains range from US$58.3bn for the wealthiest Q5 households to US$8.0bn for the poorest Q1 households. The wealthier Q5 household income gains are again driven by substantial capital income gains (US$35.1bn) and large labour income gains (US$23.2bn), which, in contrast to poorer Q1 households, are strongly influenced by skilled labour. Nonetheless, the poorer Q1 households would receive the largest relative income gains (0.39%) compared with wealthier Q5 households (0.16%).

In summary, our estimates suggest that, if a combined set of interventions, which conforms to our 2 component treatment interventions, can be developed and applied for the cost of US124bn, it would be approximately cost neutral and would additionally remove the suffering and loss caused by the more than 7m fatalities and more than 48 million cases of TB that would be averted. However, due to strong nonlinear effects focus should be on the intervention, which is, initially, deemed to be most achievable and cost-effective in reaching the twin goals of improved treatment of TB disease and increased prevention of TB transmission.

### NTEPI target for improvements in existing treatment implementation

Since the efficacies assumed for our simulated pan-TB treatment intervention may not be achievable in the short term, we also model an intervention with a treatment success rate of 91.5% that could be achieved without the need for new drug regimens, as outlined in the NTEPI’s revised estimates [34]. Results are presented in Table A and Figs B and C in S3 Appendix. Like the pan-TB treatment intervention, this improvement in existing treatment is expected to lower the population-wide average time loss to TB illness, and our simulated results (Table A in S3 Appendix) confirm that, at the population-wide level, individuals would, on average, spend roughly 24% to 25% less time being infectious and being on treatment after the introduction of NTEPI’s revised treatment intervention. Overall, it would save the Indian population from 30.4m person-years of TB illness and avoid 13.1m incident cases and reduce case fatalities by 2.0m during 2021–40, and it would increase the Indian population by 16.0m person-years and avoid 1.8m excess deaths.

Compared with business as usual, this 20% to 25% reduction in clinical and demographic effects is predicted to increase labour supplies and restore 5.8m unskilled and 0.9m skilled worker years to the Indian economy. Annual GDP per capita would increase by US$0.82 and cumulative 2021–40 NPV GDP would increase by US$28.4bn (equivalent to removing 19.4% of the economic disease burden). The NPV GDP impact is driven by the gain from reduced mortality-related labour losses (US$22.2bn) and to a lesser extent by NPV GDP gains from morbidity-related labour losses (US$3.6bn) and healthcare savings (US$2.6bn). Also, similar to the main pan-TB treatment scenario, the distributional impact estimates are regressive when it comes to absolute economic indicators, and, generally, progressive when it comes to clinical, demographic, and relative economic indicators (Figs B and C in S3 Appendix).

The predicted NPV GDP gains from implementing NTEPI’s revised treatment intervention (US$28.4bn) are roughly 20% smaller than from developing and implementing the pan-TB treatment regimen (US$35.3bn). There would therefore be a more limited budget available to implement NTEPI’s revised treatment intervention and still ensure cost neutrality by 2040. However, this strategy may not require the use of new, potentially more expensive, drugs while activities such as nutritional and financial support for TB patients to improve treatment outcomes may have wider benefits to the population.

Our sensitivity results furthermore confirm that the negative interaction effects, which characterised the combined pan-TB treatment and case detection improvements, also characterise the combined NTEPI treatment intervention and a 90% case detection rate scenario (see Table A in S3 Appendix). This could be a strong argument for pursuing NTEPI’s revised treatment intervention, in the short term, as long as the costs of implementation can be contained, and not exceed the estimated cost-neutral budget (US$28.4bn) by more than the amount the Indian government is willing to pay, additionally, for reducing human suffering from TB illness.

### Sensitivity of results to variations in incidence and mortality

In addition to the Baseline calibration 1 underlying the results presented above, we undertook 8 additional sensitivity re-calibrations 2–9 for each combination of WHO’s 2021 lower, central, and upper confidence intervals for Indian TB incidence and mortality from the “WHO TB burden estimates” [27]. For each of those 8 re-calibrated models, we re-simulated each of our 4 scenarios, including the disease burden scenario, the pan-TB treatment scenario, the 90% case detection scenario, and the combined pan-TB and 90% case detection scenario. The sensitivity results from simulating the 8 re-calibrated models are presented and discussed in S4 Appendix; the TB incidence and mortality rate assumptions underlying the baseline and 8 re-calibrated models are presented in Table A in S4 Appendix while the sensitivity analysis results are presented for a core set of outcome variables in Tables B–G in S4 Appendix.

Our model re-calibration sensitivity analyses in S4 Appendix demonstrate that our core disease burden and policy scenario results, including all main demographic, epidemiological, and macroeconomic indicators, were robust to the variations in baseline clinical outcomes. Most of our simulation results, measured by demographic, health, and economic indicators, varied within ±35 percent of our Baseline results and were in all cases within ±40 percent. This applied to all results from re-simulating disease burdens and policy scenarios. More importantly, when we focus more narrowly on relative policy scenario reductions in disease burdens, i.e. on percentage point reductions in disease burdens, we found no instances where results varied by more than ±9% points from our Baseline results. By the same token, we conclude that, while predicted absolute impacts, and thereby potential economic benefits from policy interventions, are sensitive to variations in baseline clinical outcomes, measurements of relative policy impacts on TB disease burdens are robust to such variations.

### Sensitivity results to TB patient pretreatment presenteeism

In Tables A and B in the S5 Appendix, we present the results of a sensitivity analysis where we re-simulate the baseline disease burden and policy scenario results, presented in Tables 1 and 2, with the additional assumption that working TB patients would be less productive, equivalent to a loss of 48 pretreatment working days. Similar to the sensitivity analyses above, we find that economic disease burdens and absolute policy impacts are fairly sensitive to accounting for pretreatment presenteeism of TB patients. Overall, macroeconomic and household income disease burdens would be likely to rise by 13% to 15% relative to the baseline results from Table 1, and the economic impacts of policy scenarios increase by roughly 12% to 14%, in line with the increases in underlying economic burdens. However, we also find that relative policy impacts are varying by at most ±2% (±1% points) and that relative policy impacts are therefore also robust to variations in productivity losses from pretreatment presenteeism. Overall, our sensitivity analyses therefore show a similar pattern to the above sensitivity analyses, i.e. we find that predicted absolute impacts, and thereby potential economic benefits from policy interventions, are sensitive to accounting for pretreatment presenteeism among TB patients, but we also find that relative policy impacts on TB disease burdens are not sensitive to such variations. Overall, we find that our relative policy impacts on disease burdens are very robust, but we also find that our measurement of absolute economic benefits of policy interventions to control the burden in India are sensitive to both baseline clinical outcomes and accounting for pretreatment presenteeism among TB patients, and this should be taken into account when analysing the impact of TB policy interventions and control strategies.

## Discussion

Our results predict that, without additional action, the future TB disease burden in India would include over 62.4m incident cases and 8.1m TB-related deaths between 2021 and 2040, leading to 7.0m excess deaths and a population-loss of 80.0m person-years, and an NPV GDP loss to the Indian economy of US$146.4bn. However, our results also show that significant reductions, in the twin health and economic disease burdens, are achievable if efforts are made to improve existing protocols, for detection or treatment, or to develop and implement new treatment regimens with increased efficacy. Specifically, we predict that an increase in case detection rates, to achieve the WHO End TB target of 90%, would reduce clinical and demographic disease burdens by 76% to 89% and reduce the NPV GDP disease burden by 82%, while the development and implementation of a new and more efficacious pan-TB treatment regimen would reduce the same burdens by respectively 25% to 31% and 24% over 2021 to 2040. Combining these interventions would produce a sub-additive effect, reducing the clinical and demographic disease burdens by 78% to 91% and reducing the macroeconomic burden by 84%. We also found that achievement of NTEPI’s targets for increased treatment efficacy, with scaling up based on existing treatment regimens, would achieve approximately 80% of the disease burden reductions when compared with the hypothetical pan-TB treatment regimen. Our headline macroeconomic NPV GDP indicators show that development and implementation of the individual interventions, in isolation, would be cost neutral to Indian society, as long as costs are kept below US$120.2bn (90% case detection rate), US$35.3bn (pan-TB treatment), US$124.2bn (combined), and US$28.4bn (NTEPI revised treatment protocol).

If the increased treatment efficacy and 90% case detection interventions were both available and ready to implement, the discussion, so far, clearly indicates that short-term focus should be on rapid roll-out and implementation of the 90% case detection intervention package, since it would allow for reaping of significant preventative gains from cutting the circular flow of TB infections, and achieve more than 95% of the potential disease burden reductions from combining the 2 interventions. More generally, our results suggest that improving case detection is likely to be more potent and yield bigger benefits than development of new drugs. However, neither of the increased treatment efficacy or 90% case detection rate interventions are fully developed and ready for implementation in India. In this sense, the above scenarios remain hypothetical, and our results speak more to planning the research and development necessary to achieve these targets. Moreover, it should be noted that the duration of the development and scale up period is key to determining the current value of pursuing more than one strategy.

To place our headline macroeconomic figures in the context of previous research, the Stop TB partnership previously estimated that less than half of the targeted funding of $2 billion per year, just $0.9 billion, was invested globally in the development of new TB diagnostics, drugs, and vaccines in 2020 [36]. Since India is responsible for 28% of the global TB burden [3], their estimated share of needed investments, over 2021 to 2040, is likely to be below the estimated benefits from implementation of either of our improved treatment interventions and dwarfed by the potential gains from achieving the 90% detection rate target.

To the best of our knowledge, with the exception of one other application of this model [17], previous CGE assessments, of TB disease impacts, have lacked the requisite epidemiological model underpinnings. The current macroeconomic assessment of the health and macroeconomic disease burden in India therefore fills a gap in the existing literature. Our analyses also differ strongly from previous economic assessments of TB which use alternative methodologies. Prior to this analysis, existing economic valuations of TB have used either a VSL or COI approach which are very different in scope and magnitude (a brief commentary of these methods is included in S6 Appendix). A VSL estimate of the TB burden in India (which assumed 10% to 20% fewer TB cases and deaths from 2020 to 2050 than our 20-year analysis) was US$6.63 trillion in 2020 prices [9], which is 35 times larger than our pecuniary macroeconomic estimate. This much larger figure highlights the significant difference between our pecuniary estimation approach, on the one hand, and valuations which assign willingness to pay estimates to both employed and dependent population members. Alternatively, the COI approach has been used to estimate total annual Indian illness-related financing needs, including government, out of pocket spending, and development assistance for TB, to be US$1.9bn in 2017, $644 per incident case [8]. It is striking, that the COI estimate, of annual per capita financing needs, amounts to less than one-quarter of our macroeconomic “NPV GDP per incident case” disease burden estimate of $2,345 (derived from Table 1). As suggested by WHO and others, this discrepancy points to the inapplicability of the COI approach for macroeconomic analysis, since it only provides a partial picture of the true macroeconomic impact of disease and excludes, for example, depleted capital accumulation, depletion in human capital, and demographic change to diminished economic growth [11], all of which are captured by our integrated model.

Of course, the accuracy of our results hinges on the validity of the assumptions used both in our model and scenario construction, which are made transparent in S1 Appendix and via in-depth discussion in the previous application of this model [17]. Our deterministic CGE model was fitted to target the long-term historical growth path of India, which, in spite of a downturn during the recent coronavirus pandemic period 2020 to 2022, have again returned to their long-term growth path potential, implying that our long-term model approach is robust to temporary variations around the long-term India growth path. Furthermore, our epidemiological model was fitted to target baseline WHO clinical health outcomes, and by conducting extensive sensitivity analyses which cover the full confidence intervals from the WHO TB burden estimates [27], we found that, while predicted absolute disease burden and policy impacts, and thereby potential economic benefits from policy interventions, are sensitive to accounting for both baseline clinical outcomes and pretreatment presenteeism among TB patients, relative policy impacts on TB disease burdens are not sensitive to such variations. Our core policy scenario results, including relative disease burden impacts on all main demographic, epidemiological, and macroeconomic indicators, are therefore robust under varying assumptions which gives cause for confidence in our conclusions.

That being said, in view of the unavoidable complexity of integrating macroeconomic-health-demographic models, we have used a parsimonious approach and therefore simplifications have been made which could be countered by refinements and enhancements in the future. Potential refinements include, for example, the extension to separate modelling of DS and MDR cases in the epidemiological sub-model, detailed disaggregation of public/private healthcare provision, modelling of economic determinants of more risk factors (currently, we only model consumption-related nutritional impacts on the “low BMI” risk factor), modelling of preventative treatments such as tuberculosis preventative treatment (TPT) or vaccination and more refined modelling of capital factor ownership by households (currently, we assume that, due to financial intermediation, capital factor ownership shares remain roughly constant). Our analysis also excludes potential mortality and morbidity due to post-TB lung sequelae. The lifetime impact of TB on health is increasingly being recognised. Recent analysis [37] suggests that including post-TB sequelae may increase the disability adjusted life years due to TB by over 70% in India. While the effects of this additional ill health on productivity are unknown, it is likely to increase our estimates of the health and macroeconomic benefits of reducing TB. Finally, our analysis could also be extended to account for additional benefits in terms of unpaid Indian household work, including the extent to which reduced TB burdens may increase household members’ (predominantly women’s) ability to perform unpaid household work such as child rearing, etc.

Going beyond our current policy reform interventions, previous research has analysed preventative interventions targeting nutritional risk factors for other illnesses in integrated CGE models, using, for example, food taxes as policy instruments [4,38]. Since interventions which target risk factors for TB directly (i.e., beyond externalities which for example result from indirect changes in nutritional intakes) have the potential not only to prevent TB illness, but also to potentially reduce other disease burdens, it would be beneficial for future research, to use similar tools to analyse such direct preventative interventions in combination with our treatment interventions. This would enable investigation of how these twin types of interventions may interact, and whether direct preventative effects may be additional. It would also be beneficial for such approaches to estimate potential health co-benefits for other illnesses.

While we see multiple future avenues for further extension, our integrated macroeconomic tool for assessment of TB disease burden and potential TB interventions, in India, constitutes a major step forward and provides much sought after macroeconomic evidence to guide Indian policymakers for both the prioritisation of, and investment in, interventions to meet treatment and prevention targets for TB disease burden reduction. While we acknowledge that the assumption of TB patients having similar infectiousness, regardless of whether they have been detected or not, may inflate the preventative externalities of our treatment interventions, our estimates are generally conservative, since they do not account for the additional health and macroeconomic benefits which would accumulate beyond the period of our interventions. As mentioned above, our analysis also excludes potential mortality and morbidity due to post-TB lung sequelae. Together with the other limitations mentioned above, this is left to be addressed in future research.

In conclusion, we present clear and conservative macroeconomic evidence that if the introduction of a new pan-TB (or NTEPI revised) treatment regimen, and expansion of TB detection rates from 63% to 90%, can be brought to reality in India, then substantially increased Indian investment in development and roll-out of these interventions would be warranted. Specifically, if the new pan-TB/NTEPI treatment regimen and expansion of TB detection rates can be developed and rolled-out, in combination, for a cost of U$123-124bn, we show that it would be approximately cost neutral and would additionally remove the suffering and loss caused by more than 7 million fatalities and more than 48 million cases of TB.

## Supporting information

S1 CHEERS Checklist CHEERS 2022 Checklist. (DOCX)

S1 Appendix Epidemiological model details. (DOCX)

S2 Appendix Assumptions for work absence and cost. (DOCX)

S3 Appendix Tables and figures: NTEP target for improvements in existing treatment. (DOCX)

S4 Appendix Sensitivity analysis: Variations in incidence and mortality. (DOCX)

S5 Appendix Sensitivity analysis: TB patient presenteeism. (DOCX)

S6 Appendix Existing economic methodologies. (DOCX)

## Abbreviations

BMI: body mass index
CGE: computable general equilibrium
COI: cost of illness
DR: drug-resistant
DS: drug-susceptible
GDP: gross domestic product
IFPRI: International Food Policy Research Institute
MDR: multidrug-resistant
NFHS: National Family Health Survey
NPV: net present value
SAM: Social Accounting Matrix
SEIR: susceptible–exposed–infectious–recovered
SES: socioeconomic status
TB: tuberculosis
TPT: tuberculosis preventative treatment
TRP: target regimen profile
UN: United Nations
VSL: value of statistical life
WHO: World Health Organisation

## References

[1] GBD Diseases and Injuries Collaborators 2020, Global burden of 369 diseases and injuries in 204 countries and territories, 1990–2019: a systematic analysis for the Global Burden of Disease Study 2019. Lancet. 2020;396(10258):1204–1222. DOI: 10.1016/S0140-6736(20)30925-9 PMC7567026 33069326

[2] Silva S, Arinaminpathy N, Atun R, Goosby E, Reid M. Economic impact of tuberculosis mortality in 120 countries and the cost of not achieving the Sustainable Development Goals tuberculosis targets: a full-income analysis. Lancet Glob Health. 2021;9(10):e1372–e1379. doi: 10.1016/S2214-109X(21)00299-0 34487685 PMC8415897

[3] WHO. Global tuberculosis report 2022. Geneva, World Health Organization., 2022. Available from: https://www.who.int/teams/global-tuberculosis-programme/tb-reports/global-tuberculosis-report-2022 (accessed 25 Apr 2024).

[4] Jensen HT, Keogh-Brown MR, Shankar B, Aekplakorn W, Basu S, Cuevas S, et. al. International trade, dietary change, and cardiovascular disease health outcomes: Import tariff reform using an integrated macroeconomic, environmental and health modelling framework for Thailand. SSM Popul Health. 2019;9:100435. doi: 10.1016/j.ssmph.2019.100435 31649995 PMC6804685

[5] Verguet S, Riumallo-Herl C, Gomez GB, Menzies NA, Houben RMGJ, Sumner T, et al. Catastrophic costs potentially averted by tuberculosis control in India and South Africa: a modelling study. Lancet Glob Health. 2017;5(11):e1123–e1132. doi: 10.1016/S2214-109X(17)30341-8 29025634 PMC5640802

[6] Uplekar M, Weil D, Lonnroth K, Jaramillo E, Lienhardt C, Dias HM, et. al. WHO’s new end TB strategy. Lancet. 2015;385(9979):1799–1801. doi: 10.1016/S0140-6736(15)60570-0 25814376

[7] Floyd K, Glaziou P, Houben RMGJ, Sumner T, White RG, Raviglione M. Global tuberculosis targets and milestones set for 2016–2035: definition and rationale. Int J Tuberc Lung Dis. 2018;22(7):723–730. doi: 10.5588/ijtld.17.0835 29914597 PMC6005124

[8] Yanfang S, Baena IC, Harle AC, Crosby SW, Micah AE, Siroka A, et al. Tracking total spending on tuberculosis by source and function in 135 low-income and middle-income countries, 2000–17: a financial modelling study. Lancet Infect Dis. 2020;20(8):929–942. doi: 10.1016/S1473-3099(20)30124-9 32334658 PMC7649746

[9] Silva S, Awad S, Abu-Raddad L, Atun R, Goosby E, Reid M. The Health and Economic Benefits Possible with Novel Tuberculosis Vaccines–A Modeling Study in India and Indonesia. Res Sq Pre-print. 2021. doi: 10.21203/rs.3.rs-265017/v1

[10] Bloom DE, Kuhn M, Prettner K. Modern infectious diseases: macroeconomic impacts and policy responses. J Econ Lit. 2022;60(1):85–131. doi: 10.1257/jel.20201642

[11] WHO. WHO guide to identifying the economic consequences of disease and injury. Geneva: World Health Organization, 2009. Available from: https://www.who.int/publications/i/item/9789241598293 (accessed 28 Oct 2024).

[12] Fofana MO, Knight GM, Gomez GB, White RG, Dowdy DW. Population-Level Impact of Shorter-Course Regimens for Tuberculosis: A Model-Based Analysis. PLoS ONE. 2014;9(5):e96389. doi: 10.1371/journal.pone.0096389 24816692 PMC4015982

[13] Houben RMGJ, Lalli M, Sumner T, Hamilton M, Pedrazzoli D, Bonsu F, et al. TIME Impact–a new user-friendly tuberculosis (TB) model to inform TB policy decisions. BMC Med. 2016;14(1):56. doi: 10.1186/s12916-016-0608-4 27012808 PMC4806495

[14] Mandal S, Bhatia V, Sharma M, Mandal PP, Arinaminpathy N. The potential impact of preventive therapy against tuberculosis in the WHO South-East Asian Region: a modelling approach. BMC Med. 2020;18(1):163. doi: 10.1186/s12916-020-01651-5 32684164 PMC7369473

[15] Menzies NA, Cohen T, Lin HH, Murray M, Salomon JA. Population Health Impact and Cost-Effectiveness of Tuberculosis Diagnosis with Xpert MTB/RIF: A Dynamic Simulation and Economic Evaluation. PLoS Med. 2012;9(11):e1001347. doi: 10.1371/journal.pmed.1001347 23185139 PMC3502465

[16] GOV.UK. HMRC’s CGE model documentation. Technical documents and research based on HM Revenue & Customs (HMRC) CGE model, 2013. Available from: https://www.gov.uk/government/publications/computable-general-equilibrium-cge-modelling (accessed 28 Oct 2024).

[17] Jensen HT, Keogh-Brown MR, Vassal A, Sumner T. International trade, dietary change, and TB control in India: Application of a fully-integrated macroeconomic-epidemiological model framework. GTAP Resource. 2023;6621. Available from: https://www.gtap.agecon.purdue.edu/resources/res_display.asp?RecordID=6621 (accessed 28 Oct 2024).

[18] Löfgren H, Lee Harris R, Robinson S. A Standard Computable General Equilibrium (CGE) Model in GAMS. Microcomputers and Policy Research 5, Washington DC: International Food Policy Research Institute. 2002. Available from: https://ebrary.ifpri.org/digital/collection/p15738coll2/id/74766 (accessed 26 Jul 2021).

[19] Aguiar A, Chepeliev M, Corong EL, McDougall R, van der Mensbrugghe D. The GTAP Data Base: Version 10. J Glob Econ Anal. 2019;4(1):1–27. doi: 10.21642/JGEA.040101AF

[20] Feenstra RC, Inklaar R, Timmer MP. The Next Generation of the Penn World Table Am Econ Rev. 2015;105(10):3150–3182. doi: 10.1257/aer.20130954

[21] NSSO. National Sample Survey (NSS) 68th round 2011–12, in Ministry of Statistics & Programme Implementation. National Sample Survey Office, Government of India, 2018. Available from: https://microdata.gov.in/nada43/index.php/catalog/127/study-description (accessed 15 Apr 2022).

[22] WB. World Development Indicators (WDI) database (Electronic data). 2021. Washington DC: World Bank. Available from: https://databank.worldbank.org/source/world-development-indicators (accessed 26 Jul 2021).

[23] UN. World Population Prospects, the 2019 Revision (Electronic data). 2019. New York: United Nations. Available from: http://esa.un.org/unpd/wpp/index.htm (accessed 11 Jun 2020).

[24] Fofana MO, Shrestha S, Knight GM, Cohen T, White RG, Cobelens F, et al. A Multistrain Mathematical Model To Investigate the Role of Pyrazinamide in the Emergence of Extensively Drug-Resistant Tuberculosis. Antimicrob Agents Chemother. 2017;61(3):e00498–16. doi: 10.1128/AAC.00498-16 27956422 PMC5328532

[25] IIPS. National Family Health Survey (NFHS-4), 2015–16. Mumbai: International Institute for Population Sciences (IIPS). 2017. Available from: https://dhsprogram.com/publications/publication-fr339-dhs-final-reports.cfm (accessed 28 Oct 2024).

[26] Oxlade O, Murray M. Tuberculosis and poverty: why are the poor at greater risk in India? PLoS ONE. 2012;7(1):e47533. doi: 10.1371/journal.pone.0047533 23185241 PMC3501509

[27] WHO. Data provided by countries to WHO and estimates of TB burden generated by WHO for the Global Tuberculosis Report, in Global Tuberculosis Programme (Electronic Data). 2022. Geneva: World Health Organization. Available from: https://www.who.int/teams/global-tuberculosis-programme/tb-reports/global-tuberculosis-report-2022 (accessed 25 Apr 2024).

[28] WHO. Tuberculosis Profile: India. Geneva: World Health Oganization, 2021. Available from: https://worldhealthorg.shinyapps.io/tb_profiles/?_inputs_&entity_type=%22country%22&lan=%22EN%22&iso2=%22IN%22 (accessed 25 Apr 2024).

[29] Sweeney S, Cunnama L, Laurence Y, Garcia Baena I, Kairu A, Minyewelet M, et al. Value TB Dataset: costs per intervention (Electronic Data). 2021. Harvard Dataverse. doi: 10.7910/DVN/QOI6IR; (accessed 8 Mar 2022).

[30] Arinaminpathy N, Gomez GB, Sachdeva KS, Rao R, Parmar M, Nair SA, et al. The potential deployment of a pan-tuberculosis drug regimen in India: A modelling analysis. PLoS ONE. 2020;15(3):e0230808. doi: 10.1371/journal.pone.0230808 32218585 PMC7100958

[31] WHO. Target regimen profiles for TB treatment: candidates: rifampicin-susceptible, rifampicinresistant and pan-TB treatment regimens. Geneva: World Health Organization, 2016. Available from: https://www.who.int/publications/i/item/9789241511339 (accessed 28 Oct 2024).

[32] WHO. Global tuberculosis report 2021. Geneva: World Health Organization, 2021. Available from: https://www.who.int/publications/i/item/9789240037021 (accessed 28 Oct 2024).

[33] WHO. The end TB strategy. Geneva: World Health Organization, 2015. Available from: https://www.who.int/teams/global-tuberculosis-programme/the-end-tb-strategy (accessed 28 Oct 2024).

[34] RNTCP. National Strategic Plan for Tuberculosis Elimination 2017–2025. Revised National Tuberculosis Control Programme, Central TB Division, Ministry of Health with Family Welfare, Nirman Bhavan, New Delhi, 2017. Available from: https://tbcindia-wp.azurewebsites.net/national-strategic-plan-2017-2025-for-tb-elimination-in-india/ (accessed 28 Oct 2024).

[35] Sinha P, Carwile M, Bhargava ACintron C, Acuna-Villaorduna C, Lakshminarayan S, et al. How much do Indians pay for tuberculosis treatment? A cost analysis. Public Health Action. 2020;10(3):110–17. doi: 10.5588/pha.20.0017 33134125 PMC7577002

[36] TAG. Stop TB Partnership. Tuberculosis research funding trends 2005–2020. New York: Treatment Action Group, 2021. Available from: https://www.stoptb.org/tuberculosis-research-funding-trends-2005-2021-0 (accessed 28 Oct 2024).

[37] Menzies NA, Quaife M, Allwood BW, Byrne AL, Coussens AK, Harries AD, et al. Lifetime burden of disease due to incident tuberculosis: a global reappraisal including post-tuberculosis sequelae. Lancet Glob Health. 2021;9(12):e1679–e1687. doi: 10.1016/S2214-109X(21)00367-3 34798027 PMC8609280

[38] Jensen HT, Keogh-Brown MR, Shankar B, Aekplakorn W, Basu S, Cuevas S, et al. Palm oil and dietary change: application of an integrated macroeconomic, environmental, demographic, and health modelling framework for Thailand. Food Policy. 2019;83:92–103. doi: 10.1016/j.foodpol.2018.12.003 31007358 PMC6472326

