# Supplementary material for: Estimating the health and macroeconomic burdens of tuberculosis in India, 2021–2040: A fully integrated modelling study
Source: PLoS Med. 2024 Dec 12;21(12):e1004491. doi: 10.1371/journal.pmed.1004491 (PMC11637336; doi:10.1371/journal.pmed.1004491)
Supplement: S1 Appendix — (DOCX) [file pmed.1004491.s002.docx]

# S1 Appendix. Epidemiological model details

**Supplement to:**

Estimating the health and macroeconomic burdens of tuberculosis in India, 2021-2040: A fully-integrated modelling study

**Authors:**

Marcus R. Keogh-Brown, Tom Sumner, Sedona Sweeney, Anna Vassall, Henning Tarp Jensen,

**Correspondence:**

Marcus Keogh-Brown

Faculty of Public Health and Policy

London School of Hygiene & Tropical Medicine

London

UK

Email: marcus.keogh-brown@lshtm.ac.uk

The epidemiological model simulates a population of fixed size (N = 1) with births and deaths and tracks the proportion of the population in each health state over time. The key outputs of the epidemiological model used by the demographic and macroeconomic models are incident TB cases, deaths due to TB and TB treatment initiations. These are scaled to the true population size and distributed by age and sex in the demographic model. Full details of the integration of the epidemiological model into the framework are given in [1].

The core structure of the TB model is shown in figure A with state and parameter definitions given in tables B and C. Individuals in the susceptible state (S) may be infected (at rate λ, which depends on the number of infectious individuals). A proportion (α) progress to TB disease (I), the remainder (1-α) enter a latent infection state (L). Individuals in L may progress to TB disease due to reactivation (at rate c) or following re-infection (at rate pλ). Infectious individuals may self-cure (at rate ε) be diagnosed and start treatment (T) (at rate d) or die due to TB (at rate m). Individuals complete treatment at rate ν. A proportion τ are successfully treated and return to the latent state (L). For the remainder (1-τ), treatment is unsuccessful. To keep track of notifications of new incident TB cases we include separate compartments (Ir and Tr) for individuals who have already been treated for their current episode of TB. Individuals in Ir are infectious and contribute to the force of infection (see below).

The model is stratified into five household types representing wealth quintiles, with each household type represented by an instance of the core model and linked via the transmission of mycobacterium between household types (see below for details).

To facilitate linkage with the CGE model the TB model is implemented as a set of difference equations given below.

Details of the parameter values, stratification by wealth quintiles, model fitting and improved diagnosis scenario reported in the main text are given on subsequent pages.

**
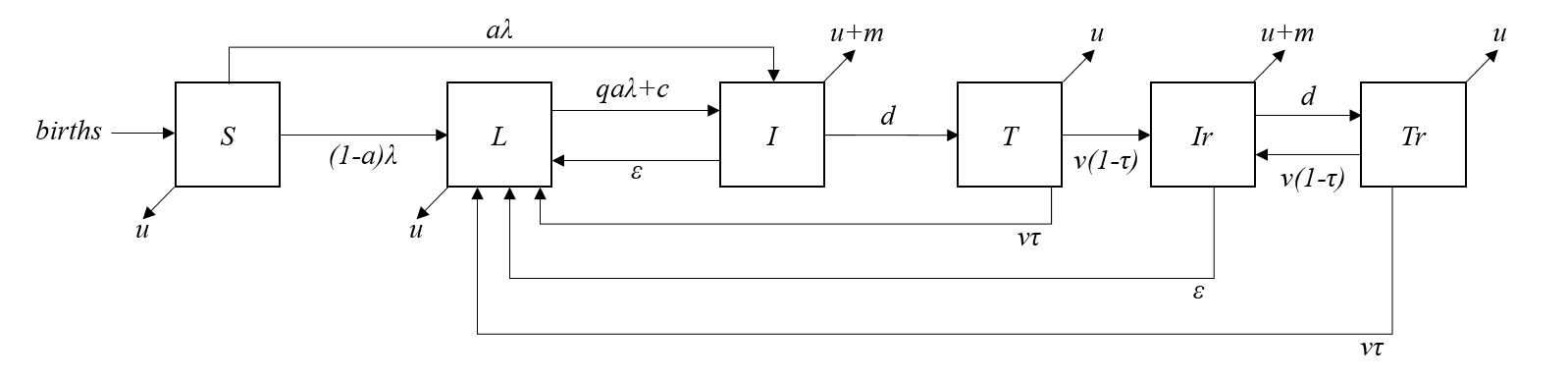
Figure A.** Core TB model structure. Improved diagnostics will increase the rate of transition (d) from I (Ir) to T (Tr). Improved treatment will increase the proportion (τ) who experience successful treatment and move from T (Tr) to L (and reduce the proportion moving from T (Tr) to Ir).

| S | Susceptible, never exposed to M.tb. |
| --- | --- |
| L | Latently, infected with M.tb, not infectious |
| I | TB disease, infectious |
| Ir | TB disease, infectious, previously had treatment for this episode |
| T | On treatment for TB disease |
| Tr | On treatment for TB disease, previously had treatment for this episode |
| N | Total population |

**Table B.** State definitions in the TB model

| U | background mortality rate |
| --- | --- |
| M | excess mortality rate due to active TB |
| A | proportion progressing directly to disease on infection* |
| C | risk of developing active TB from latent infection* |
| Q | relative risk of active TB following re-infection |
| Ε | risk of self-cure of active TB |
| D | risk of diagnosis of active TB |
| Τ | proportion successfully treated |
| V | 1/average duration of treatment |
| β_in_ | transmission risk between those in same SES quintile |
| β_rel_ | relative transmission risk between SES quintiles |

**Table C.** Parameter definitions in the TB model. Parameters indicated with * vary by household type.

**Difference equations**

In the following, Subscript *i* = 1, … ,5 indicates wealth quintile with 1 being the lowest and 5 the highest wealth group.

Susceptible, S (describes the infection of susceptible individuals and the birth and death process)

$$S_{i,t+1}=S_{i,t}-\lambda_{i,t}S_{i,t}+u_{i,t}\left( N_{i,t}-S_{i,t} \right)+m_{i,t}\left( I_{i,t}+{Ir}_{i,t} \right)$$

Latently infected, L (describes the progression of latently infected individuals to disease and the recovery (via slef-cure or treatment) of infectious individuals)

$$L_{i,t+1}=L_{i,t}+\left( 1-a_{i,t} \right)\lambda_{i,t}S_{i,t}-\left( qa_{i,t}\lambda_{i,t}+c_{i,t}+u_{i,t} \right)L_{i,t}+\varepsilon\left( I_{i,t}+{Ir}_{i,t} \right)+v\tau_{i,t}{(T}_{i,t}+{Tr}_{i,t})$$

TB disease, I (describes the progression to TB disease, and the diagnosis, self-cure of death of people with TB)

$$I_{i,t+1}=I_{i,t}+a_{i,t}\lambda_{i,t}S_{i,t}+\left( qa_{i,t}\lambda_{i,t}+c_{i,t} \right)L_{i,t}-\left( d_{i,t}+\varepsilon+u_{i,t}+m_{i,t} \right)I_{i,t}$$

TB disease (previously had treatment for this episode), Ir (as above but tracking individuals who have previously been treated for their current episode of TB)

$${Ir}_{i,t+1}={Ir}_{i,t}+v\left( 1-\tau_{i,t} \right){(T}_{i,t}+{Tr}_{i,t})-\left( d_{i,t}+\varepsilon+u_{i,t}+m_{i,t} \right){Ir}_{i,t}$$

On TB treatment, T (describes the initiation and completion of treatment for people with TB)

$$T_{i,t+1}=T_{i,t}+d_{i,t}I_{i,t}-(v+u_{i,t})T_{i,t}$$

On TB retreatment (previously had treatment for this episode), Tr (as above but tracking individuals who have previously been treated for their current episode of TB)

$${Tr}_{i,t+1}={Tr}_{i,t}+d_{i,t}{Ir}_{i,t}-(v+u_{i,t}){Tr}_{i,t}$$

**Other expressions**

Total population (the total population in the model is the sum of all states)

$$N_{i,t}=S_{i,t}+L_{i,t}+I_{i,t}+{Ir}_{i,t}+T_{i,t}+{Tr}_{i,t}$$

Force of infection

The force of infection depends on the number of infectious individuals in the population (this includes those who have been previously treated for their current episode of TB (Ir)) and the rates of effective contact between individuals. We assume that individuals make effective contact with others in the same wealth quintile at a rate β_in_ and with those in all other quintiles at a reduced rate given by β_rel_ x β_in_. The force of infection in quintile i is then given by:

$$\lambda_{i,t}=\beta_{in}\left( \left( I_{i,t}+{Ir}_{i,t} \right)+\beta_{rel}\sum_{j\neq i} \left( I_{j,t}+{Ir}_{j,t} \right) \right)$$

TB incidence (counts individuals developing TB disease, excludes those failing treatment who are not classed as new incident cases)

$${Inc}_{i,t}= a_{i,t}\lambda_{i,t}S_{i,t}+\left( qa_{i,t}\lambda_{i,t}+c_{i,t} \right)L_{i,t}$$

TB prevalence (includes anyone with prevalent TB (irrespective of treatment history)

$${Prev}_{i,t}=I_{i,t}+{Ir}_{i,t}$$

TB notifications (diagnosed and started on TB treatment) (only includes those starting treatment for the first time for their current episode of TB)

$${Notif}_{i,t}=d_{i,t}I_{i,t}$$

TB mortality (deaths occurring among people with TB disease (irrespective of treatment history)

$${Mort}_{i,t}=m_{i,t}{(I}_{i,t}+{Ir}_{i,t})$$

**Parameter values**

| **Symbol** | **Definition** | **Value/constraints for fitting** | **Units** | **Source** |
| --- | --- | --- | --- | --- |
| a_0_ | Proportion progressing directly to disease on infection | 0.084 | Dimensionless | Value in absence of risk factors. Based on analysis to reproduce cumulative incidence of TB following exposure [2, 3]^$^ |
| q | Relative risk of active TB following re-infection | 0.6 | Dimensionless | Assumes 40% protection against disease from re-infection due to prior exposure [4] |
| τ | Proportion successfully treated | 0.8 | Dimensionless | Weighted average of public and private sector treatment outcomes and DS and MDR (see below for derivation) |
| β_in_ | Effective contact between those in same wealth quintile | >0 | per week | Estimated from fitting of model to data |
| β_out_ | Relative effective contact between wealth quintiles | 0-1 | Dimensionless | Estimated from fitting of model to data |
| u | Background mortality | 3.8454e-4 | per week | Assumes average life-expectancy of 50 years |
| m | Excess mortality due to active TB | >0 | per week | Estimated from fitting of model to data |
| ε | Self-cure of active TB | >0 | per week | Estimated from fitting of model to data |
| c_0_ | Developing active TB from latent infection | 1.1423e-5 | per week | Value in absence of risk factors. Based on analysis to reproduce cumulative incidence of TB following exposure [2, 3]^$^ |
| d | Diagnosis of active TB | >0 | per week | Estimated from fitting of model to data |
| v | Completion of treatment | 3.7731e-2 | per week | Assuming average treatment duration of 6 months |

**Table D**. Parameter values. ^$^values of a and c in wealth quintiles are calculated based on risk factors (see below)

**Adjustment of notifications and treatment outcomes**

Our simplified model does not explicitly include the mix of private and public sector TB care in India or a dynamic representation of drug resistant TB. To account for these simplifications we made the following adjustments to the reported notifications and treatment outcome data.

Notifications

Notification data represents the reported number of people started on TB treatment. However, notification data reported to WHO by the India national TB programme does not capture all cases treated in the private sector. Fitting to the reported number of notifications may therefore underestimate the diagnostic rate. Given the proportion of cases started on treatment that are included in the notification data, *P*, we can adjust the notification data to give the “true” number started on treatment (the number entering the T state in the model).

Assuming that all public sector treatments are notified, then:

$$\begin{aligned} P=P_{T,PUB}+{x.P}_{T,PRI}\# \end{aligned}$$

Where *P_T,PUB_* = 0.6 is the proportion of all TB patients treated in the public sector, *P_T,PRI_* = 0.4 [5] is the proportion treated in the private sector and the unknown *x* is the proportion of private sector treatment that is notified. We have an estimate that *P_N,PRI_* = 0.3 of all TB notifications came from the private sector in 2020 [6]. We can write this as:

$$\begin{aligned} P_{N,PRI}=\frac{x.P_{T,PRI}}{x.P_{T,PRI}+P_{T,PUB}}\# \end{aligned}$$

and rearrange to give an expression for *x*:

$$\begin{aligned} x=\frac{P_{N,PRI}P_{T,PUB}}{P_{T,PRI}\left( 1-P_{N,PRI} \right)}=\frac{0.3\times0,6}{0.4 \left( 1-0.3 \right)}=0.64\# \end{aligned}$$

which we can then use to give P = 0.85.

Treatment outcomes

Arinaminpathy et al [7] use data from Uplekar et al [8] to inform the proportion of individuals completing first line treatment in the private sector: τ_PRIV_ = 0.6 (0.4-0.8). Treatment success reported by WHO is *τ_WHO_* = 0.84. Assuming that this includes both public and private sector treatment we can write:$\begin{aligned} \tau_{WHO}=\left( 1-P_{N,PRI} \right)\tau_{PUB}+P_{N,PRI}\tau_{PRI}\# \end{aligned}$

which can be rearranged to give the treatment success in the public sector

$$\begin{aligned} \tau_{PUB}=\frac{\tau_{WHO}-P_{N,PRI}\tau_{PRI}}{1-P_{N,PRI}}=\frac{0.84-0.3\times0.6}{1-0.3}=0.94\# \end{aligned}$$

and the weighted average treatment success, τ is:

$$\begin{aligned} \tau=\tau_{PRIV}P_{T,PRI}+\tau_{PUB}P_{T,PUB}=\left( 0.6\times0.4 \right)+\left( 0.94\times0.6 \right)=0.81\# \end{aligned}$$

Approximately 3% of treated TB cases are being treated for multi-drug resistant (MDR) TB. Assuming that no MDR treatment is given in the private sector and using an MDR treatment success rate of 56% (WHO reported values) we can calculate the final weighted treatment success $\begin{aligned} \tau=\left( 0.03\times0.56 \right)+\left( 0.97\times0.81 \right)=0.80\# \end{aligned}$

**TB risks by SES**

The differences in TB risk by SES are likely mediated by several factors. Using the approach in Andrews et al [9] we can use the prevalence of these risk factors and the relative risks for each factor to calculate the relative parameters for disease progression (*a* and *c*) across different wealth quintiles. This assumes the values of a and c given in table above represent those in the absence of risk factors.

Assuming we can represent the relationship between the risk of TB (if exposed) and the risk factors using a poisson model, the log risk of TB in SES strata *i* is given by:

$\ln\left( z_{i} \right)=\alpha+\sum_{j} g_{j}p_{ij}$

Where *g_j_* is the **log** of the relative risk (RR) for risk factor *j* and *p_ij_* is the prevalence of risk factor *j* in strata *i*. The risk rate ratio for TB among strata *i* compared to a population with no risk factors is:

$${RR}_{i}=exp(\ln\left( z_{i} \right)-\ln\left( z_{0} \right))$$

Because p_ij_ = 0 in the absence of risk factors, ln(z_0_) = α and therefore:

$${RR}_{i}=exp(\sum_{j} g_{j}p_{ij})$$

Based on Oxlade et al [10] we assuming the following risk factors shown in table E contribute to the increased risk of TB with declining wealth quintile. Using data on the prevalence of these risk factors from the NFHS-4 and estimates of their relative risks for TB from Oxlade et al [10] we estimate the overall risk for developing TB by wealth quintile shown in the final row of table E. These multipliers are used to adjust the parameters a and c in each wealth quintile-based strata of the model.

|  | **Prevalence of risk factor by wealth quintile** | | | | |  |
| --- | --- | --- | --- | --- | --- | --- |
| **Risk factor** | **1** | **2** | **3** | **4** | **5** | **RR for risk factor** |
| Smoking (any current) | 17.9 | 16.3 | 14.15 | 12.35 | 9.65 | 2.0 |
| Indoor air pollution (use of biomasss cooking fuel inside the home) | 79.1 | 68.5 | 51 | 25.2 | 8.3 | 1.4 |
| Low BMI (<18.5) | 33.9 | 28.1 | 21.8 | 16.7 | 11.1 | 2.1 |
| Alcohol (daily) | 3.1 | 2.2 | 1.9 | 1.3 | 0.98 | 2.9 |
| Diabetes (self-report) | 0.5 | 0.7 | 0.9 | 1.3 | 2.0 | 3.1 |
| HIV (testing in DHS) | 0.4 | 0.4 | 0.5 | 0.4 | 0.2 | 26.7 |
| **RR for overall risk of TB** | **2.0008** | **1.8156** | **1.6133** | **1.3993** | **1.2421** |  |

**Table E**. Risk factor prevalence from Indian NFHS-4 and RRs from Oxlade et al.

**Model fitting**

The model was fitted to WHO estimates of TB incidence and TB mortality, TB notifications (adjusted for private sector treatment (see above)) and TB prevalence overall and by wealth quintile (see table F).

Fitting was carried out using a simple least squares minimisation process, implemented using the Nelder-Mead algorithm as implemented in the FME package in R.

We allowed the diagnostic rate (d), the self-cure rate (ε), the TB mortality rate (m), and the within and between quintile (B_in_, B_rel_) contact rates to vary.

To account for uncertainty in the data, we fitted the model to the midpoint and the low and high estimates of TB incidence and mortality (and all possible combinations). Table G shows the fitted values for d, ε, m B_in_ and B_rel_

| **Variable** | | **Observed** |
| --- | --- | --- |
| TB incidence (WHO) | | 200 (172-230) |
| TB mortality (WHO) | | 26 (19-34) |
| TB notifications (adjusted for private sector) | | 140 |
| Prevalence (India National Health Survey NFHS-4) | 1 | 497 |
|  | 2 | 365 |
|  | 3 | 301 |
|  | 4 | 234 |
|  | 5 | 148 |
| Prevalence | | 301 |

**Table F.** Model fitting targets. All values are per 100,000 population.

| **TB Incidence** | **TB Mortality** | **d** | **ε** | **m** | **B_in_** | **B_rel_** | **d’** |
| --- | --- | --- | --- | --- | --- | --- | --- |
| 200 | 26 | 0.010128 | 0.002356 | 0.001627 | 0.282878 | 0.072259 | 0.038585 |
| 200 | 19 | 0.010128 | 0.002794 | 0.001189 | 0.283985 | 0.072051 | 0.038585 |
| 200 | 34 | 0.010128 | 0.001857 | 0.002127 | 0.284763 | 0.072497 | 0.03859 |
| 172 | 26 | 0.009995 | 0.001109 | 0.00162 | 0.250502 | 0.075032 | 0.027658 |
| 172 | 19 | 0.010125 | 0.001337 | 0.001188 | 0.24806 | 0.075129 | 0.025861 |
| 172 | 34 | 0.009943 | 0.00071 | 0.002114 | 0.251833 | 0.075004 | 0.028488 |
| 230 | 26 | 0.009919 | 0.004393 | 0.001627 | 0.328784 | 0.069538 | 0.056105 |
| 230 | 19 | 0.009922 | 0.004826 | 0.001189 | 0.328942 | 0.069338 | 0.056077 |
| 230 | 34 | 0.009921 | 0.003889 | 0.002127 | 0.328502 | 0.069791 | 0.056063 |

**Table G.** Fitted parameter values corresponding to each combination of incidence and mortality

**Implementation of improved diagnosis scenario**

In the main text we present results from a scenario in which the proportion of incident TB cases in India which are diagnosed and started on treatment (the case detection rate, CDR) is increased from 63% to 90%. We implement this improvement in the model through a change in the diagnostic rate, *d*.

The baseline value of d is estimated via model fitting (see above). The increased value of the diagnostic rate, *d’* is calculated as follows:

In the model there are 4 possible outcomes for incident cases: diagnosis (*d’*), self-cure (*e*), natural mortality (*u*), excess TB mortality (*m*). The proportion of incidence cases that are diagnosed and started on treatment (CDR) is given by:

$$CDR=\frac{d'}{d'+u+e+m}$$

which can be rearranged to give:

$$d'=\frac{CDR(e+u+m}{1-CDR}$$

We note that this approach results in a short term large increase in the number of notifications and as the diagnostic rate is applied to the population of prevalent TB cases but gives the target CDR in the longer term.

Values of d’ corresponding to each set of fitted parameters are given in table G above.

References

1. Jensen HT, Keogh-Brown MR, Vassal A, Sumner T. *International trade, dietary change, and TB control in India: Application of a fully-integrated macroeconomic-epidemiological model framework.* GTAP Resource 6621, 2023. URL: <https://www.gtap.agecon.purdue.edu/resources/res_display.asp?RecordID=6621>. (accessed 28. October 2024)

2. Menzies NA, Cohen T, Lin HH, Murray M, Salomon JA. *Population Health Impact and Cost-Effectiveness of Tuberculosis Diagnosis with Xpert MTB/RIF: A Dynamic Simulation and Economic Evaluation.* **PLoS** Med, 2012. **9**(11):e1001347. DOI: <https://doi.org/10.1371/journal.pmed.1001347>.

3. Sumner T, Jensen HT, Keogh-Brown MR, Vassall A. *Time to integrate epidemiological and economic models for TB.* Int J Tuberc Lung Dis, 2022. **26**(3):282-284. DOI: <https://doi.org/10.5588/ijtld.21.0448>.

4. Vynnycky E, Fine PEM. *The natural history of tuberculosis: the implications of age-dependent risks of disease and the role of reinfection.* Epidemiol Infect, 1997. **119**(2):183-201. DOI: <https://doi.org/10.5588/ijtld.21.044810.1017/S0950268897007917>.

5. NSSO. *National Sample Survey (NSS) 68th round 2011-12*, in Ministry of Statistics & Programme Implementation. National Sample Survey Office, Government of India, 2018. URL: https://microdata.gov.in/nada43/index.php/catalog/127/study-description. (accessed 15. April 2022)

6. WHO. *Contribution of public-private mix to TB case notifications in priority countries, 2010–2020 (Figure 3.1.8)*, in Global tuberculosis report 2021. Geneva: World Health Organization, 2021. URL: <https://www.who.int/publications/digital/global-tuberculosis-report-2021/tb-diagnosis-treatment/notifications#fig--3-1-8>.

7. Arinaminpathy N, Gomez GB, Sachdeva KS, Rao R, Parmar M, Nair SA, et al. *The potential deployment of a pan-tuberculosis drug regimen in India: A modelling analysis.* PLoS One, 2020. **15**(3):e0230808. DOI: <https://doi.org/10.1371/journal.pone.0230808>.

8. Uplekar M, Weil D, Lonnroth K, Jaramillo E, Lienhardt C, Dias HM, et al. *WHO's new end TB strategy.* Lancet, 2015. **385**(9979):1799-1801. DOI: <https://doi.org/10.1016/S0140-6736(15)60570-0>.

9. Andrews JR, Basu S, Dowdy DW, Murray MB. *The epidemiological advantage of preferential targeting of tuberculosis control at the poor.* Int J Tuberc Lung Dis, 2015. **19**(4):375-80. DOI: <https://doi.org/10.5588/ijtld.14.0423>.

10. Oxlade O, Murray M. *Tuberculosis and poverty: why are the poor at greater risk in India?* PLoS One, 2012. **7**(1):e47533. DOI: <https://doi.org/10.1371/journal.pone.0047533>.
