# Supplementary material for: Estimating the health and macroeconomic burdens of tuberculosis in India, 2021–2040: A fully integrated modelling study
Source: PLoS Med. 2024 Dec 12;21(12):e1004491. doi: 10.1371/journal.pmed.1004491 (PMC11637336; doi:10.1371/journal.pmed.1004491)
Supplement: S2 Appendix — (DOCX) [file pmed.1004491.s003.docx]

## S2 Appendix. Assumptions for work absence and cost

**Supplement to:**

Estimating the health and macroeconomic burdens of tuberculosis in India, 2021-2040: A fully-integrated modelling study

**Authors:**

Marcus R. Keogh-Brown, Tom Sumner, Sedona Sweeney, Anna Vassall, Henning Tarp Jensen,

**Correspondence:**

Marcus Keogh-Brown

Faculty of Public Health and Policy

London School of Hygiene & Tropical Medicine

London

UK

Email: marcus.keogh-brown@lshtm.ac.uk

**Assumption 1: Share of MDR Cases: 3%**

We assume that 3% of all TB cases are MDR. The WHO TB profile for India [1] states that in 2021 India had:

2116976 notified TB cases

58837 Lab conf MDR/RR

We round the resulting estimate of 2.8% MDR cases to 3%

**Assumption 2: DS-TB Costs: US136.15 (2018 US$)**

Cost of TS-TB treatment is based on adult pulmonary TB for both new and relapse cases, taken from [2] and adjusted to 2021 prices (the base year of the model). **US136.15 (2018 US$)**

**Assumption 3: DR -TB costs: US1,229.29 (2018 US$)**

We assume a total DR-TB cost per case of **US1,229.29 (2018 US$)**, taken from [2] and adjusted to 2021 prices (the base year of the model).

**Assumption 4: Absence from work**

Using data from figure 1 of Chatterjee et. al 2023 [3] we derive the difference in employment due to TB to be 36.2% for the intensive treatment phase and 31.7% for the continuation phase. Furthermore, we assume from [4] that the intensive and continuation phases for DS-TB are 2 and 4 months respectively, and that the DR-TB treatment has an intensive phase of 6 months and a continuation phase of zero. Multiplying the phase durations by the employment percentages and assuming 22.7 working days per month (5 working days * 52 weeks / 12 months) we derive work absence durations of 8.6 weeks and 9.4 weeks for DS-TB and DR-TB respectively.

References

1. WHO. *Tuberculosis Profile: India*. Geneva: World Health Oganization, 2021; URL: <https://worldhealthorg.shinyapps.io/tb_profiles/?_inputs_&entity_type=%22country%22&lan=%22EN%22&iso2=%22IN%22>. (accessed 25. April 2024)

2. Sweeney S, Cunnama L, Laurence Y, Garcia Baena I, Kairu A, Minyewelet M, et al. *Value TB Dataset: costs per intervention (Electronic Data).* 2021. Harvard Dataverse. DOI: <https://doi.org/10.7910/DVN/QOI6IR>. (accessed 8. March 2022).

3. Chatterjee S, Das P, Shikhule A, Munje R, Vassall A. *Journey of the tuberculosis patients in India from onset of symptom till one-year post-treatment.* PLOS Glob Public Health, 2023. **3**(2):e0001564. DOI: <https://doi.org/10.1371/journal.pgph.0001564>.

4. Chatterjee S, Das P, Vassall A. *Impact of COVID-19 restrictive measures on income and health service utilization of tuberculosis patients in India.* BMC Infect Dis, 2022. **22**(1):711. DOI: <https://doi.org/10.1186/s12879-022-07681-z>.
