# Supplementary material for: Estimating the health and macroeconomic burdens of tuberculosis in India, 2021–2040: A fully integrated modelling study
Source: PLoS Med. 2024 Dec 12;21(12):e1004491. doi: 10.1371/journal.pmed.1004491 (PMC11637336; doi:10.1371/journal.pmed.1004491)
Supplement: S3 Appendix — (DOCX) [file pmed.1004491.s004.docx]

## S3 Appendix. Tables and Figures: NTEP Target for Improvements in existing treatment implementation

**Supplement to:**

Estimating the health and macroeconomic burdens of tuberculosis in India, 2021-2040: A fully-integrated modelling study

**Authors:**

Marcus R. Keogh-Brown, Tom Sumner, Sedona Sweeney, Anna Vassall, Henning Tarp Jensen,

**Correspondence:**

Marcus Keogh-Brown

Faculty of Public Health and Policy

London School of Hygiene & Tropical Medicine

London

UK

Email: marcus.keogh-brown@lshtm.ac.uk

| **Table A 2021-2040 Estimated GDP and final demand impacts - SENSITIVITY NTEPI treatment (reduced eff) and 90% case detection scenarios (USD/bn USD), (Δ = change relative to business as usual, #person-years = number of person years, NPV = Net Present Value, hhld = Household, # = 'number of')** | | | | | | | | | | | | | | |
| --- | --- | --- | --- | --- | --- | --- | --- | --- | --- | --- | --- | --- | --- | --- |
|  | Disease Burden (DB*) | |  | Scenario: NTEPI treatment | | |  | Scenario: 90% case detection | | |  | Scenario: combined | | |
| Indicators |  |  |  |  |  |  |  |  |  |  |  |  |  |  |
| ECONOMIC |  |  |  |  |  |  |  |  |  |  |  |  |  |  |
| GDP PER CAPITA (USD) | USD | % change |  | USD | % change | % of DB* |  | USD | % change | % of DB* |  | USD | % change | % of DB* |
| - ∆Real GDP/capita/year | 4.21 | 0.217% |  | 0.82 | 0.042% | 19.5% |  | 3.47 | 0.179% | 82.4% |  | 3.56 | 0.184% | 84.6% |
| NPV of GDP (2021-2040) (bn USD) | bn USD | % change |  | bn USD | % change | % of DB* |  | bn USD | % change | % of DB* |  | bn USD | % change | % of DB* |
| - ∆NPV GDP | -146.4 | -0.215% |  | 28.4 | 0.042% | -19.4% |  | 120.2 | 0.176% | -82.1% |  | 123.4 | 0.181% | -84.3% |
| - ∆NPV GDP (Treatment Costs) | -15.7 | -0.023% |  | 2.6 | 0.004% | -16.6% |  | 11.2 | 0.016% | -71.0% |  | 11.5 | 0.017% | -72.9% |
| - ∆NPV GDP (∆Labour supply - Morbidity) | -18.4 | -0.027% |  | 3.6 | 0.005% | -19.4% |  | 13.4 | 0.020% | -72.9% |  | 13.7 | 0.020% | -74.4% |
| - ∆NPV GDP (∆Labour supply - Mortality) | -113.3 | -0.166% |  | 22.2 | 0.033% | -19.6% |  | 95.6 | 0.140% | -84.3% |  | 98.2 | 0.144% | -86.6% |
| NPV of hhld income and consumption (2021-2040) (bn USD) | bn USD | % change |  | bn USD | % change | % of DB* |  | bn USD | % change | % of DB* |  | bn USD | % change | % of DB* |
| - ∆NPV Household income | -116.8 | -0.171% |  | 22.6 | 0.039% | -19.4% |  | 95.9 | 0.166% | -82.1% |  | 98.4 | 0.171% | -84.3% |
| - ∆NPV Labour income | -66.7 | -0.098% |  | 12.8 | 0.034% | -19.2% |  | 54.6 | 0.144% | -81.9% |  | 56.1 | 0.148% | -84.1% |
| - ∆NPV Capital income | -50.1 | -0.073% |  | 9.8 | 0.050% | -19.6% |  | 41.3 | 0.208% | -82.4% |  | 42.3 | 0.214% | -84.5% |
| - ∆NPV Household consumption | -102.2 | -0.150% |  | 19.8 | 0.046% | -19.4% |  | 83.9 | 0.194% | -82.1% |  | 86.1 | 0.199% | -84.3% |
| Labour market (1000s person-years) | 1000s | % change |  | 1000s | % change | % of DB* |  | 1000s | % change | % of DB* |  | 1000s | % change | % of DB* |
| - ∆Unskilled labour (#person-years) | -28,627.7 | -0.313% |  | 5,783 | 0.063% | -20.2% |  | 24,026 | 0.263% | -83.9% |  | 24,641 | 0.270% | -86.1% |
| - ∆Skilled labour (#person-years) | -4,496.3 | -0.222% |  | 898 | 0.044% | -20.0% |  | 3,756 | 0.186% | -83.5% |  | 3,854 | 0.191% | -85.7% |
| **CLINICAL, EPIDEMIOLOGICAL & DEMOGRAPHIC** |  |  |  |  |  |  |  |  |  |  |  |  |  |  |
| Demographic outcomes | 1000s | % of total |  | 1000s | % of total | % of DB* |  | 1000s | % of total | % of DB* |  | 1000s | % of total | % of DB* |
| - ∆Population (1000s prs-years) | -80,029.5 | -0.27% |  | 16,000 | 0.05% | -20.0% |  | 68,114 | 0.23% | -85.1% |  | 69,921 | 0.23% | -87.4% |
| - Excess deaths (1000s persons) | 7,042.4 | 3.58% |  | -1,777 | -0.90% | -25.2% |  | -6,274 | -3.19% | -89.1% |  | -6,395 | -3.25% | -90.8% |
| Clinical Outcomes (1000 persons) | 1000s | % change |  | 1000s | % change | % of DB* |  | 1000s | % change | % of DB* |  | 1000s | % change | % of DB* |
| - ∆TB incident cases | 62,424 | - |  | -13,144 | -21.1% | -21.1% |  | -47,272 | -75.7% | -75.7% |  | -48,219 | -77.2% | -77.2% |
| - ∆TB case fatalities | 8,102 | - |  | -2,047 | -25.3% | -25.3% |  | -7,208 | -89.0% | -89.0% |  | -7,348 | -90.7% | -90.7% |
| Cumulative population by compartment (mm pers-yrs†) | mm | % change |  | mm | % change | % of DB* |  | mm | % change | % of DB* |  | mm | % change | % of DB* |
| - ∆Susceptible population | 18,239.3 | - |  | 444.3 | 2.4% | 2.4% |  | 1,955.2 | 10.7% | 10.7% |  | 2,008.5 | 11.0% | 11.0% |
| - ∆Latently infected population | 11,703.2 | - |  | -397.9 | -3.4% | -3.4% |  | -1,787.6 | -15.3% | -15.3% |  | -1,835.8 | -15.7% | -15.7% |
| - ∆Infectious population ‡ | 95.8 | - |  | -24.2 | -25.3% | -25.3% |  | -85.2 | -89.0% | -89.0% |  | -86.8 | -90.7% | -90.7% |
| - ∆Treatment population $ | 25.4 | - |  | -6.2 | -24.4% | -24.4% |  | -14.2 | -56.1% | -56.1% |  | -15.9 | -62.6% | -62.6% |
| Risk factors (endogenous) | %-points | % change |  | %-points | % change | % of DB* |  | %-points | % change | % of DB* |  | %-points | % change | % of DB* |
| - ∆low BMI prevalence (avg) | 20.45% | - |  | -0.25% | -1.2% | -1.2% |  | -1.04% | -5.1% | -5.1% |  | -1.07% | -5.2% | -5.2% |
| Memorandum Items (2021-2040): |  |  |  |  |  |  |  |  |  |  |  |  |  |  |
| - Real GDP/capita/year (USD) | 1,939 |  |  |  |  |  |  |  |  |  |  |  |  |  |
| - NPV GDP (bn USD) | 68,198 |  |  |  |  |  |  |  |  |  |  |  |  |  |
| Notes: Own calculations; *We use "DB" to refer to "Total Disease Burden impact" on any given economic, epidemiological, and demographic indicator; †We use "mm prs-yrs" to refer to millions of cumulative person-years over our 2021-40 time horizon; ‡ The infectious population consists of both newly infectious and previously unsuccessfully treated persons; $ The treatment population consists of both persons who are newly treated and re-treated after prior unsuccessful treatment. | | | | | | | | | | | | | | |

| **ΔTotal Household Income** | |
| --- | --- |
| bn USD | % change |
|  |  |
| **ΔHousehold Labour Income** | |
| bn USD | % change |
|  |  |
| **ΔHousehold Capital Income** | |
| bn USD | % change |
|  |  |
| Figure B. TB NTEPI treatment scenario impacts: Household income composition and distribution (Δ = change relative to business as usual) | |

| **Labour supplies by household** | |
| --- | --- |
| ΔTotal labour supplies (1000s work-years) | ΔTotal labour supplies (% changes) |
|  |  |
| ΔUnskilled labour supplies (1000s work-years) | ΔSkilled labour supplies (1000s work-years) |
|  |  |
| **Demographics** | |
| ΔPopulation (1000s person-years) | ΔExcess Deaths (1000s persons) |
|  |  |
| **Clinical outcomes** | |
| ΔTB incident cases (1000s persons) | ΔTB case fatalities (1000s persons) |
|  |  |
| Figure C. NTEPI treatment scenario impacts – Labour market, Demographics, Clinical outcomes (Δ = change relative to business as usual) | |
