# Supplementary material for: Estimating the health and macroeconomic burdens of tuberculosis in India, 2021–2040: A fully integrated modelling study
Source: PLoS Med. 2024 Dec 12;21(12):e1004491. doi: 10.1371/journal.pmed.1004491 (PMC11637336; doi:10.1371/journal.pmed.1004491)
Supplement: S4 Appendix — (DOCX) [file pmed.1004491.s005.docx]

## S4 Appendix. Sensitivity analysis: Variations in incidence and mortality

**Supplement to:**

Estimating the health and macroeconomic burdens of tuberculosis in India, 2021-2040: A fully-integrated modelling study

**Authors:**

Marcus R. Keogh-Brown, Tom Sumner, Sedona Sweeney, Anna Vassall, Henning Tarp Jensen,

**Correspondence:**

Marcus Keogh-Brown

Faculty of Public Health and Policy

London School of Hygiene & Tropical Medicine

London

UK

Email: marcus.keogh-brown@lshtm.ac.uk

WHO estimated that, in 2021, Indian TB incidence was 200 per 100,000 while Indian TB mortality was 26 per 100,000 – and WHO also indicated that the confidence interval for TB incidence was [172; 230] per 100,000 while the confidence interval for TB mortality was [19; 34] [1]. We employed the central 2021 WHO estimates of Indian TB incidence (200 per 100,000) and mortality (26 per 100,000) to calibrate our Baseline epidemiological model and for simulating our Baseline results presented in this article.

In order to test the sensitivity of our results to the adoption of the WHO’s central estimates of TB incidence (200 per 100,000) and mortality (26 per 100,000), we re-calibrated our integrated model framework to target all of the nine permutations of the aforementioned lower bound/central estimate/upper bound of 2021 Indian TB incidence (172/200/230 per 100,000) and of 2021 Indian TB mortality (19/26/34 per 100,000). This meant that, in addition to the original Baseline calibration 1, we undertook eight Sensitivity re-calibrations 2-9, and, for each of those eight re-calibrated models, we undertook re-simulation of each of our four scenarios, including our Disease Burden scenario, our pan-TB treatment scenario, our 90% case detection scenario, and our combined pan-TB treatment and 90% case detection scenario. The Baseline and eight re-calibrations, and their underlying assumptions, are presented in Table A.

Below, we report and discuss the results from the sensitivity analyses of our disease burden and policy scenario results, based on the abovementioned eight re-calibrated model frameworks. We present four key clinical, epidemiological and demographic indicators in Tables B-E and two key economic indicators in Tables F and G. Below, we discuss each indicator in their separate sub-sections, and we conclude that, while TB disease burdens and TB policy impacts vary with the underlying morbidity and mortality assumptions, our policy results are generally robust, in particular when it comes to our measures of policy-related relative reductions in disease burdens.

In what follows, we will refer to the following three groups of models as “same-mortality” groups: (calibration 1, calibration 4, calibration 7), (calibration 2, calibration 5, calibration 8), (calibration 3, calibration 6, calibration 9) (see Table A.)

In a similar way, we will refer to the following three groups of models as “same-incidence” groups: (calibration 1, calibration 2, calibration 3), (calibration 4, calibration 5, calibration 6), (calibration 7, calibration 8, calibration 9) (see Table A.)

### Demographic Indicator estimates: Population impacts

The cumulative population (POP) impacts, measured by person-years lost (PYL), of our sensitivity analyses are shown in Table B. The relative Disease Burden (DB) POP impacts vary from [-0.199%; -0.194%] (lower bound mortality re-calibrations 2, 5, 8) to [-0.348%; -0.344%] (higher bound mortality re-calibrations 3, 6, 9). This translates into 58-60mn PYLs for lower bound mortality and 104-105mn PYLs for higher bound mortality – and this roughly equates to a 25-27 percent reduction respectively a 30-31 percent increase relative to our Baseline DB POP impact (80mn PYLs). We also note that incidence bound variations do not matter very much for DB POP (biggest difference is approx. 1.5mn PYLs, between re-calibrations 5 and 8) while mortality bound variations naturally matter more (biggest difference is approx. 46.5mn PYLs, between re-calibrations 8 and 9).

Turning to our estimated policy scenario impacts on DB POP, “pan-TB treatment” leads to a robust 23.5-25.7% reduction; “90% case detection” leads to a robust 76.5-90.0% reduction; and the combined policy scenario leads to a robust 81.4%-91.8% reduction. While the relative DB POP policy effectiveness levels are robust all around, the absolute impacts of the combined policy scenario vary from 49-53mn PYLs saved for lower bound mortality to 86-96mn PYLs saved for higher bound mortality – and this roughly equates to a 24-31 percent reduction respectively a 23-37 percent increase relative to our Baseline combined scenario DB POP impact (70mn PYLs saved).

While our sensitivity analyses therefore confirm our findings that the pan-TB treatment and 90% case detection policies are effective in lowering DB POP in relative terms (and that sizes of relative policy impacts are robust), predicted absolute measures of DB POP and policy impacts naturally vary (substantially) with mortality bound variations. Hence, absolute DB POP varies from 58-60mn PYLs (lower bound mortality) to 104-105mn PYLs (higher bound mortality), while absolute combined policy impacts vary between 49-53mn PYLs saved (lower bound mortality) to 86-96mn PYLs saved (higher bound mortality).

### Demographic Indicator estimates: Excess Deaths impacts

The cumulative Excess Deaths (ED) impacts of our sensitivity analyses are measured in persons (PRSs) and shown in Table C. The DB ED impacts vary from [2.60%; 2.68%] (lower bound same-mortality group 2, 5, 8) to [4.64%; 4.69%] (higher bound same-mortality group 3, 6, 9). This translates into 5.1-5.3mn EDs for lower bound mortality and 9.1-9.2mn EDs for higher bound mortality – and these numbers equate roughly to a 25-27 percent reduction and a 30-31 percent increase relative to our estimated Baseline DB ED impact (7.0mn EDs).

We note that incidence bound variations do not matter very much for DB ED (largest “same-mortality” group difference is approx. 0.2mn EDs, between re-calibrations 5 and 8 in group (2, 5, 8)) while mortality bound variations matter more (largest “same-incidence” group difference is approx. 4.1mn EDs, between re-calibrations 8 and 9 in group (7, 8, 9)).

Turning to our estimated policy scenario impacts on DB ED, “pan-TB treatment” leads to a robust 29.0-32.3% reduction; “90% case detection” leads to a robust 82.6-92.6% reduction; and the combined policy scenario leads to a robust 86.4%-94.0% reduction. While the relative DB ED policy effectiveness levels are robust all around, the absolute impacts of the combined policy scenario vary quite strongly from 4.6-4.8mn EDs avoided for lower bound mortality to 8.0-8.6mn EDs avoided for higher bound mortality – and this roughly equates to a 25-29 percent reduction respectively a 25-35 percent increase relative to our Baseline combined scenario DB ED impact (6.4mn EDs avoided).

While our sensitivity analyses of DB ED, similar to DB POP, therefore confirm, that the pan-TB treatment and 90% case detection policies are effective in lowering DB ED in relative terms (and that sizes of relative policy impacts are robust), absolute measures of DB ED and policy impacts naturally vary (substantially) with mortality bound variations. Hence, absolute DB ED varies from 5.1-5.3mn EDs (lower bound mortality) to 9.1-9.2mn EDs (higher bound mortality), while absolute combined policy impacts vary between 4.6-4.8mn EDs avoided (lower bound mortality) to 8.0-8.6mn EDs avoided (higher bound mortality).

### Epidemiological Indicator Estimates: TB Incidence Impacts

The cumulative TB Incidence (INC) impacts of our sensitivity analyses are measured in Incident Cases (IC) and shown in Table D. The DB INC impacts vary from 56-57mn ICs for lower bound incidence and 71-72mn ICs for higher bound incidence – and this roughly equates to a 9-11 percent reduction respectively a 14-15 percent increase relative to our Baseline DB INC impact (62mn ICs).

We note that incidence bound variations matter very much for DB INC (largest “same-mortality” group difference is approx. 16mn ICs, between re-calibrations 6 and 9 in group (3, 6, 9)) while mortality bound variations matter much less (largest “same-incidence” group difference is approx. 0.9mn ICs, between re-calibrations 5 and 6 in group (4, 5, 6)).

Turning to our estimated policy scenario impacts on DB INC, “pan-TB treatment” leads to a robust 24.2-26.9% reduction; “90% case detection” leads to a robust 69.8-79.5% reduction; and the combined policy scenario leads to a robust 73.2%-80.7% reduction. While the relative DB INC policy effectiveness levels are robust all around, the absolute impacts of the combined policy scenario vary quite strongly from 41-42mn ICs avoiding TB for lower bound incidence to 57-58mn ICs avoiding TB for higher bound incidence – and these numbers roughly equate to a 14.3-14.4 percent reduction and a 18-19 percent increase relative to our Baseline combined scenario DB ED impact (48mn ICs avoiding TB illness).

While our sensitivity analyses of DB INC therefore confirm that the pan-TB treatment and 90% case detection policies are effective in lowering DB INC in relative terms (and that sizes of relative policy impacts are robust), absolute measures of DB INC and policy impacts naturally vary (substantially) with incidence bound variations. Hence, absolute DB INC varies from 56-57mn ICs (lower bound mortality) to 71-72mn ICs (higher bound mortality), while absolute combined policy impacts vary between 41-42mn ICs avoiding TB (lower bound mortality) to 57-58mn ICs avoiding TB (higher bound mortality).

### Epidemiological Indicator Estimates: TB Case Fatalities Impacts

The cumulative TB Case Fatalities (CF) impacts of our sensitivity analyses are measured in CFs and shown in Table E. The DB CF impacts vary from 5.9-6.1mn CFs for lower bound mortality to 10.5-10.6mn CFs for higher bound mortality – and these numbers roughly equate to a 25-27 percent reduction and a 30-31 percent increase relative to our Baseline DB CF impact (8.1mn CFs).

We note that incidence bound variations matter very little for DB CF (largest “same-mortality” group difference is approx. 0.2mn CFs between re-calibrations 5 and 8 in group (2, 5, 8)) while mortality bound variations matter much more (largest “same-incidence” group difference is approx. 4.7mn CFs between re-calibrations 8 and 9 in group (7, 8, 9)).

Turning to policy scenario impacts on DB CF, “pan-TB treatment” leads to a robust 29.0-32.4% reduction; “90% case detection” leads to a robust 82.4-92.6% reduction; and the combined policy scenario leads to a robust 86.2%-93.9% reduction. While the relative DB CF policy effectiveness levels are robust all around, the absolute impacts of the combined policy scenario (similar to the DB POP, DB ED, and DB INC impacts above) vary quite strongly from 5.2-5.5mn CFs avoided for lower bound mortality to 9.2-9.9mn CFs avoided for higher bound mortality – and this roughly equates to a 25-29 percent reduction respectively a 25-35 percent increase relative to our Baseline combined scenario DB CF impact (7.4mn CFs avoided).

While our sensitivity analyses therefore confirm that the pan-TB treatment and 90% case detection policies are effective in lowering DB CF in relative terms (and that sizes of relative policy impacts are robust), absolute measures of DB CF and policy impacts naturally vary (substantially) with mortality bound variations. Hence, absolute DB CF varies from 5.9-6.1mn CFs (lower bound mortality) to 10.5-10.6mn CFs (higher bound mortality), while absolute combined policy impacts vary between 5.2-5.5mn CFs avoided (lower bound mortality) to 9.2-9.9mn CFs avoided (higher bound mortality).

### Economic Indicator Estimates: Real GDP per capita Impacts

The cumulative Real GDP per capita (RGDPcap) impacts of our sensitivity analyses are shown in Table F. The DB RGDPcap impacts vary from 3.30-3.46USD for lower bound mortality to 5.07-5.36USD for higher bound mortality – and these numbers roughly equate to an 18-22 percent reduction and a 20-27 percent increase relative to our Baseline DB RGDPcap impact (4.21USD).

We note that incidence bound variations matter less for DB RGDPcap (largest “same-mortality” group difference is approx. 0.29USD between re-calibrations 6 and 9 in group (3, 6, 9)) while mortality bound variations seem to matter considerably more (largest “same-incidence” group difference is approx. 1.89USD between re-calibrations 8 and 9 in group (7, 8, 9)) but, as commented at the end of this sub-section, this points to the possibility that, even though incidence bound variations may not be important for DB RGDPcap assessment, these variations may play a more important role for assessment of DB RGDPcap policy impacts.

Turning to policy scenario impacts on DB RGDPcap, “pan-TB treatment” leads to a robust 22.8-25.1% reduction; “90% case detection” leads to a robust 73.8-87.4% reduction; and the combined policy scenario leads to a robust 78.4%-89.2% reduction. While the relative DB RGDPcap policy effectiveness levels are robust all around, the absolute impacts of the combined policy scenario vary quite strongly from 2.59-3.03USD costs avoided, for lower bound mortality, to 4.12-4.78USD costs avoided, for higher bound mortality – and these numbers roughly equate to a 15-28 percent reduction and a 15-33 percent increase relative to our Baseline combined scenario DB RGDPcap impact (3.59USD costs avoided).

While our sensitivity analyses of DB RGDPcap therefore confirm that the pan-TB treatment and 90% case detection policies are effective in lowering DB RGDPcap in relative terms (and that sizes of relative policy impacts are robust), absolute measures of DB RGDPcap and policy impacts naturally vary (substantially) with mortality bound variations. Hence, absolute DB RGDPcap varies from 3.30-3.46USD (lower bound mortality) to 5.07-5.36USD (higher bound mortality), while absolute combined policy impacts vary between 2.59-3.03USD costs avoided (lower bound mortality) to 4.12-4.78USD costs avoided (higher bound mortality).

In terms of the relative importance of mortality vs. incidence bound variation, we also note that, while relative combined policy impacts on DB RGDPcap are robust, the combined policy impacts have relatively wide ranges including (1) 2.59-3.03USD costs avoided with a range of 0.44USD (for lower bound mortality), and (2) 4.12-4.78USD costs avoided with a range of 0.66USD (for higher bound mortality), and these relatively wide DB RGDPcap impact ranges indicate that not only mortality bound variation but also incidence bound variation is important for the combined policy impacts on DB RGDPcap, i.e. that economic outcome measures are sensitive not only to mortality assumptions, but to some extent also to incidence assumptions (which makes sense since mortality is known to have critically important economic snowballing effects, but the high numbers of incident TB cases also means that, in spite of smaller per person costs, these costs will add up to a noticeable part of the overall economic TB burden, and interventions targeted at reducing the number of infectious individuals, and thereby reducing the number of incident cases, should therefore exhibit noticeable sensitivity to both mortality and incidence bound variations).

### Economic Indicator Estimates: NPV GDP Impacts

The NPV of GDP (NPVGDP) impacts of our sensitivity analyses are shown in Table G. The DB NPVGDP impacts vary from 115-120bn USD for lower bound mortality to 176-186bn USD for higher bound mortality – and these numbers roughly equate to an 18-22 percent reduction respectively a 20-27 percent increase relative to our Baseline DB NPVGDP impact (146bn USD).

We note that incidence bound variations do not matter very much for DB NPVGDP (largest “same-mortality” group difference is approx. 10bn USD between re-calibrations 6 and 9 in group (3, 6, 9)) while mortality bound variations matter much more (largest “same-incidence” group difference is approx. 66bn USD between re-calibrations 8 and 9 in group (7, 8, 9)).

Turning to policy scenario impacts on DB NPVGDP, “pan-TB treatment” leads to a robust 22.6-25.0% reduction; “90% case detection” leads to a robust 73.6-87.1% reduction; and the combined policy scenario leads to a robust 78.1%-88.9% reduction. While the relative DB NPVGDP policy effectiveness levels are robust all around, the absolute impacts of the combined policy scenario vary quite strongly from 90-105bn USD costs avoided, for lower bound mortality, to 143-165bn USD costs avoided, for higher bound mortality – and these numbers roughly equate to a 15-28 percent reduction and a 15-33 percent increase relative to our Baseline combined scenario DB NPVGDP impact (124bn USD avoided).

While our sensitivity analyses of DB NPVGDP therefore confirm that the pan-TB treatment and 90% case detection policies are effective in lowering DB NPVGDP in relative terms (and that sizes of relative policy impacts are robust), absolute measures of DB NPVGDP and policy impacts naturally vary (substantially) with mortality bound variations. Hence, absolute DB NPVGDP varies from 115-120bn USD (lower bound mortality) to 176-186bn USD (higher bound mortality), while absolute combined policy impacts vary between 90-105bn USD costs avoided (lower bound mortality) to 143-165bn USD costs avoided (higher bound mortality).

In terms of the relative importance of mortality vs. incidence bound variation, we note, similar to DB RGDPcap, that the combined policy impacts have relatively wide ranges including (1) 90-115bn USD costs avoided with a range of 15bn USD (for lower bound mortality), and (2) 143-165bn USD costs avoided with a range of 22bn USD (for higher bound mortality), and these relatively wide DB NPVGDP impact ranges indicate, similar to DB RGDPcap, that not only mortality bound variation but also incidence bound variation is important for the combined policy impacts on DB NPVGDP.

### Sensitivity analysis summary

In summary, we have demonstrated that our core disease burden and policy scenario results, including all main demographic, epidemiological, and macroeconomic indicators of our disease burden and policy simulations, were robust to variations in model re-calibration focussed on capturing the full confidence intervals from the “WHO TB burden estimates” on 2021 Indian TB incidence (200[172;230] per 100k) and on 2021 Indian TB mortality (26[19;34] per 100k) [1]. We calibrated our Baseline model to the central estimates ((incidence, mortality) = (200 per 100k, 26 per 100k)), and then re-calibrated our model to the eight other perturbations of lower bound, central estimate, and upper bound for respectively Indian TB incidence and Indian TB mortality from 2021 (see Table A).

We found that variations in baseline assumptions about TB incidence and mortality rates within the WHOs sensitivity intervals for 2021 Indian TB incidence and mortality rates [1], meant that most of our simulation results, measured by demographic, health, and economic indicators, varied within ±35 percent of our Baseline results (and all varied within ±40 percent). This applied to all results from re-simulating disease burdens and re-simulating our policy scenarios. More importantly, we found that, when we focused narrowly on measuring impacts on disease burden reductions, i.e. focusing on those indicators which are normalized by the indicator-specific disease burden, we found no instances of sensitivity analyses where results varied by more than 15 percentage points from each other – and, critically, we found no instances where any sensitivity analyses varied by more than ±10 percentage points from our Baseline results. This result demonstrates that most of the absolute variations in policy impacts, which we observe in our sensitivity analyses, stem from underlying changes in disease burdens, due to the re-calibration of our epidemiological model. By the same token, we can confirm that our results are robust both when it comes to absolute outcomes and especially when it comes to our measurements of relative impact reductions on TB disease burdens.

| **Table A. Sensitivity analyses - WHO confidence intervals around 2021 Indian TB incidence and mortality rates - model re-calibrations and scenario re-simulations** | | | | | | | | |
| --- | --- | --- | --- | --- | --- | --- | --- | --- |
|  |  |  | Incidence |  |  |  | Mortality* |  |
|  |  | Lower Bound | Central Estimate | Upper Bound |  | Lower Bound | Central Estimate | Upper Bound |
| Baseline | Calibration 1 |  | 200 |  |  |  | 26 |  |
| Sensitivity Scenarios | Calibration 2 |  | 200 |  |  | 19 |  |  |
|  | Calibration 3 |  | 200 |  |  |  |  | 34 |
|  | Calibration 4 | 172 |  |  |  |  | 26 |  |
|  | Calibration 5 | 172 |  |  |  | 19 |  |  |
|  | Calibration 6 | 172 |  |  |  |  |  | 34 |
|  | Calibration 7 |  |  | 230 |  |  | 26 |  |
|  | Calibration 8 |  |  | 230 |  | 19 |  |  |
|  | Calibration 9 |  |  | 230 |  |  |  | 34 |
| Notes: *The 2021 Indian TB mortality estimates have later been revised downwards to a central estimate of 25 per 100,000 and a confidence interval of [18;33] | | | | | | | | |

| **Table B. 2021-2040 Demographic outcomes: Population - sensitivity impacts - pan-TB treatment and 90% case detection scenarios** | | | | | | | | | | | | | | |
| --- | --- | --- | --- | --- | --- | --- | --- | --- | --- | --- | --- | --- | --- | --- |
|  | Disease Burden (DB*) | |  | Scenario: pan-TB treatment | | |  | Scenario: 90% case detection | | |  | Scenario: combined | | |
| Indicators |  |  |  |  |  |  |  |  |  |  |  |  |  |  |
| CLINICAL, EPIDEMIOLOGICAL & DEMOGRAPHIC |  |  |  |  |  |  |  |  |  |  |  |  |  |  |
| - ∆Population (1000s prs-years) | 1000s | % of total |  | 1000s | % of total | % of DB* |  | 1000s | % of total | % of DB* |  | 1000s | % of total | % of DB* |
| Baseline calibration 1 | -80,029.54 | -0.266% |  | 19,890.95 | 0.066% | -24.9% |  | 68,113.57 | 0.227% | -85.1% |  | 70,375.62 | 0.234% | -87.9% |
| Sensitivity calibration 2 | -58,431.17 | -0.194% |  | 14,478.81 | 0.048% | -24.8% |  | 49,703.67 | 0.165% | -85.1% |  | 51,359.33 | 0.171% | -87.9% |
| Sensitivity calibration 3 | -104,771.68 | -0.348% |  | 26,132.75 | 0.087% | -24.9% |  | 89,230.76 | 0.297% | -85.2% |  | 92,183.36 | 0.307% | -88.0% |
| Sensitivity calibration 4 | -80,094.56 | -0.266% |  | 20,326.20 | 0.068% | -25.4% |  | 62,863.52 | 0.209% | -78.5% |  | 66,324.85 | 0.221% | -82.8% |
| Sensitivity calibration 5 | -59,707.33 | -0.199% |  | 15,373.49 | 0.051% | -25.7% |  | 45,698.23 | 0.152% | -76.5% |  | 48,571.96 | 0.162% | -81.4% |
| Sensitivity calibration 6 | -103,566.24 | -0.344% |  | 26,122.98 | 0.087% | -25.2% |  | 82,112.35 | 0.273% | -79.3% |  | 86,387.09 | 0.287% | -83.4% |
| Sensitivity calibration 7 | -79,787.56 | -0.265% |  | 18,785.17 | 0.062% | -23.5% |  | 71,744.54 | 0.239% | -89.9% |  | 73,193.43 | 0.243% | -91.7% |
| Sensitivity calibration 8 | -58,211.90 | -0.194% |  | 13,663.45 | 0.045% | -23.5% |  | 52,319.35 | 0.174% | -89.9% |  | 53,380.36 | 0.178% | -91.7% |
| Sensitivity calibration 9 | -104,693.55 | -0.348% |  | 24,762.51 | 0.082% | -23.7% |  | 94,181.83 | 0.313% | -90.0% |  | 96,077.17 | 0.320% | -91.8% |
| Notes: Own calculations; *We use "DB" to refer to "Total Disease Burden impact" on any given economic, epidemiological, and demographic indicators | | | | | | | | | | | | | | |

| **Table C. 2021-2040 Demographic outcomes: Excess Deaths - sensitivity impacts - pan-TB treatment and 90% case detection scenarios** | | | | | | | | | | | | | | |
| --- | --- | --- | --- | --- | --- | --- | --- | --- | --- | --- | --- | --- | --- | --- |
|  | Disease Burden (DB*) | |  | Scenario: pan-TB treatment | | |  | Scenario: 90% case detection | | |  | Scenario: combined | | |
| Indicators |  |  |  |  |  |  |  |  |  |  |  |  |  |  |
| CLINICAL, EPIDEMIOLOGICAL & DEMOGRAPHIC |  |  |  |  |  |  |  |  |  |  |  |  |  |  |
| '- Excess deaths (1000s persons) | 1000s | % of total |  | 1000s | % of total | % of DB* |  | 1000s | % of total | % of DB* |  | 1000s | % of total | % of DB* |
| Baseline calibration 1 | 7,042.38 | 3.582% |  | -2,182.36 | -1.110% | -31.0% |  | -6,273.69 | -3.191% | -89.1% |  | -6,424.91 | -3.268% | -91.2% |
| Sensitivity calibration 2 | 5,141.08 | 2.615% |  | -1,586.20 | -0.807% | -30.9% |  | -4,577.12 | -2.328% | -89.0% |  | -4,688.01 | -2.384% | -91.2% |
| Sensitivity calibration 3 | 9,221.16 | 4.690% |  | -2,871.95 | -1.461% | -31.1% |  | -8,220.56 | -4.181% | -89.1% |  | -8,417.54 | -4.281% | -91.3% |
| Sensitivity calibration 4 | 7,059.84 | 3.591% |  | -2,250.94 | -1.145% | -31.9% |  | -5,936.75 | -3.019% | -84.1% |  | -6,176.12 | -3.141% | -87.5% |
| Sensitivity calibration 5 | 5,272.48 | 2.682% |  | -1,704.56 | -0.867% | -32.3% |  | -4,353.02 | -2.214% | -82.6% |  | -4,554.53 | -2.316% | -86.4% |
| Sensitivity calibration 6 | 9,120.28 | 4.639% |  | -2,890.87 | -1.470% | -31.7% |  | -7,726.98 | -3.930% | -84.7% |  | -8,020.71 | -4.079% | -87.9% |
| Sensitivity calibration 7 | 7,010.12 | 3.565% |  | -2,041.69 | -1.038% | -29.1% |  | -6,491.00 | -3.301% | -92.6% |  | -6,586.36 | -3.350% | -94.0% |
| Sensitivity calibration 8 | 5,113.63 | 2.601% |  | -1,482.49 | -0.754% | -29.0% |  | -4,732.68 | -2.407% | -92.6% |  | -4,802.64 | -2.443% | -93.9% |
| Sensitivity calibration 9 | 9,201.18 | 4.680% |  | -2,696.90 | -1.372% | -29.3% |  | -8,524.07 | -4.335% | -92.6% |  | -8,648.55 | -4.399% | -94.0% |
| Notes: Own calculations; *We use "DB" to refer to "Total Disease Burden impact" on any given economic, epidemiological, and demographic indicator | | | | | | | | | | | | | | |

| **Table D. 2021-2040 Clinical Outcomes: TB Incident Cases - sensitivity impacts - pan-TB treatment and 90% case detection scenarios** | | | | | | | | | | | | | | |
| --- | --- | --- | --- | --- | --- | --- | --- | --- | --- | --- | --- | --- | --- | --- |
|  | Disease Burden (DB*) | |  | Scenario: pan-TB treatment | | |  | Scenario: 90% case detection | | |  | Scenario: combined | | |
| Indicators |  |  |  |  |  |  |  |  |  |  |  |  |  |  |
| CLINICAL, EPIDEMIOLOGICAL & DEMOGRAPHIC |  |  |  |  |  |  |  |  |  |  |  |  |  |  |
| - ∆TB incident cases (1000 persons) | 1000s | % of total |  | 1000s | % of total | % of DB* |  | 1000s | % of total | % of DB* |  | 1000s | % of total | % of DB* |
| Baseline calibration 1 | 62,424.03 | - |  | -16,158.02 | -25.884% | -25.9% |  | -47,271.59 | -75.727% | -75.7% |  | -48,454.92 | -77.622% | -77.6% |
| Sensitivity calibration 2 | 62,337.15 | - |  | -16,017.96 | -25.696% | -25.7% |  | -47,106.20 | -75.567% | -75.6% |  | -48,293.15 | -77.471% | -77.5% |
| Sensitivity calibration 3 | 62,529.82 | - |  | -16,321.47 | -26.102% | -26.1% |  | -47,466.97 | -75.911% | -75.9% |  | -48,646.35 | -77.797% | -77.8% |
| Sensitivity calibration 4 | 56,070.00 | - |  | -14,901.43 | -26.576% | -26.6% |  | -39,846.67 | -71.066% | -71.1% |  | -41,507.97 | -74.029% | -74.0% |
| Sensitivity calibration 5 | 56,675.55 | - |  | -15,270.96 | -26.945% | -26.9% |  | -39,566.22 | -69.812% | -69.8% |  | -41,461.84 | -73.156% | -73.2% |
| Sensitivity calibration 6 | 55,762.66 | - |  | -14,738.55 | -26.431% | -26.4% |  | -39,946.14 | -71.636% | -71.6% |  | -41,513.97 | -74.448% | -74.4% |
| Sensitivity calibration 7 | 71,359.84 | - |  | -17,421.73 | -24.414% | -24.4% |  | -56,593.01 | -79.307% | -79.3% |  | -57,458.97 | -80.520% | -80.5% |
| Sensitivity calibration 8 | 71,194.39 | - |  | -17,250.08 | -24.230% | -24.2% |  | -56,355.81 | -79.158% | -79.2% |  | -57,224.79 | -80.378% | -80.4% |
| Sensitivity calibration 9 | 71,642.70 | - |  | -17,665.05 | -24.657% | -24.7% |  | -56,947.97 | -79.489% | -79.5% |  | -57,812.85 | -80.696% | -80.7% |
| Notes: Own calculations; *We use "DB" to refer to "Total Disease Burden impact" on any given economic, epidemiological, and demographic indicators | | | | | | | | | | | | | | |

| **Table E. 2021-2040 Clinical Outcomes: TB Cases Fatalities - sensitivity impacts - pan-TB treatment and 90% case detection scenarios** | | | | | | | | | | | | | | |
| --- | --- | --- | --- | --- | --- | --- | --- | --- | --- | --- | --- | --- | --- | --- |
|  | Disease Burden (DB*) | |  | Scenario: pan-TB treatment | | |  | Scenario: 90% case detection | | |  | Scenario: combined | | |
| Indicators |  |  |  |  |  |  |  |  |  |  |  |  |  |  |
| CLINICAL, EPIDEMIOLOGICAL & DEMOGRAPHIC |  |  |  |  |  |  |  |  |  |  |  |  |  |  |
| - ∆TB case fatalities | 1000s | % of total |  | 1000s | % of total | % of DB* |  | 1000s | % of total | % of DB* |  | 1000s | % of total | % of DB* |
| Baseline calibration 1 | 8,101.94 | - |  | -2,512.11 | -31.006% | -31.0% |  | -7,207.54 | -88.961% | -89.0% |  | -7,382.80 | -91.124% | -91.1% |
| Sensitivity calibration 2 | 5,914.55 | - |  | -1,825.53 | -30.865% | -30.9% |  | -5,258.39 | -88.906% | -88.9% |  | -5,386.90 | -91.079% | -91.1% |
| Sensitivity calibration 3 | 10,608.57 | - |  | -3,306.61 | -31.169% | -31.2% |  | -9,444.29 | -89.025% | -89.0% |  | -9,672.59 | -91.177% | -91.2% |
| Sensitivity calibration 4 | 8,122.68 | - |  | -2,592.72 | -31.920% | -31.9% |  | -6,817.58 | -83.933% | -83.9% |  | -7,094.10 | -87.337% | -87.3% |
| Sensitivity calibration 5 | 6,066.47 | - |  | -1,963.34 | -32.364% | -32.4% |  | -4,998.58 | -82.397% | -82.4% |  | -5,231.13 | -86.230% | -86.2% |
| Sensitivity calibration 6 | 10,493.13 | - |  | -3,329.94 | -31.734% | -31.7% |  | -8,873.68 | -84.567% | -84.6% |  | -9,213.13 | -87.802% | -87.8% |
| Sensitivity calibration 7 | 8,064.19 | - |  | -2,348.62 | -29.124% | -29.1% |  | -7,460.00 | -92.508% | -92.5% |  | -7,570.72 | -93.881% | -93.9% |
| Sensitivity calibration 8 | 5,882.52 | - |  | -1,704.99 | -28.984% | -29.0% |  | -5,439.17 | -92.463% | -92.5% |  | -5,520.39 | -93.844% | -93.8% |
| Sensitivity calibration 9 | 10,584.74 | - |  | -3,103.10 | -29.317% | -29.3% |  | -9,796.62 | -92.554% | -92.6% |  | -9,941.17 | -93.920% | -93.9% |
| Notes: Own calculations; *We use "DB" to refer to "Total Disease Burden impact" on any given economic, epidemiological, and demographic indicator | | | | | | | | | | | | | | |

| **Table F. 2021-2040 REAL GDP PER CAPITA - sensitivity impacts - pan-TB treatment and 90% case detection scenarios (USD/bn USD)** | | | | | | | | | | | | | | |
| --- | --- | --- | --- | --- | --- | --- | --- | --- | --- | --- | --- | --- | --- | --- |
|  | Disease Burden (DB*) | |  | Scenario: pan-TB treatment | | |  | Scenario: 90% case detection | | |  | Scenario: combined | | |
|  |  |  |  |  |  |  |  |  |  |  |  |  |  |  |
| GDP PER CAPITA | USD | % change |  | USD | % change | % of DB |  | USD | % change | % of DB |  | USD | % change | % of DB |
| - ∆Real GDP/capita/year (USD) |  |  |  |  |  |  |  |  |  |  |  |  |  |  |
| Baseline calibration 1 | -4.21 | -0.217% |  | 1.02 | 0.053% | -24.3% |  | 3.47 | 0.179% | -82.4% |  | 3.59 | 0.185% | -85.1% |
| Sensitivity calibration 2 | -3.33 | -0.172% |  | 0.80 | 0.041% | -24.1% |  | 2.72 | 0.140% | -81.6% |  | 2.81 | 0.145% | -84.3% |
| Sensitivity calibration 3 | -5.22 | -0.269% |  | 1.27 | 0.066% | -24.4% |  | 4.33 | 0.223% | -83.0% |  | 4.47 | 0.231% | -85.7% |
| Sensitivity calibration 4 | -4.12 | -0.212% |  | 1.02 | 0.053% | -24.8% |  | 3.14 | 0.162% | -76.2% |  | 3.31 | 0.171% | -80.4% |
| Sensitivity calibration 5 | -3.30 | -0.170% |  | 0.83 | 0.043% | -25.1% |  | 2.44 | 0.126% | -73.8% |  | 2.59 | 0.133% | -78.4% |
| Sensitivity calibration 6 | -5.07 | -0.261% |  | 1.05 | 0.054% | -20.8% |  | 3.92 | 0.202% | -77.4% |  | 4.12 | 0.213% | -81.4% |
| Sensitivity calibration 7 | -4.34 | -0.224% |  | 1.00 | 0.051% | -22.9% |  | 3.77 | 0.194% | -86.8% |  | 3.84 | 0.198% | -88.5% |
| Sensitivity calibration 8 | -3.46 | -0.179% |  | 0.79 | 0.041% | -22.8% |  | 2.98 | 0.153% | -85.9% |  | 3.03 | 0.156% | -87.6% |
| Sensitivity calibration 9 | -5.36 | -0.276% |  | 1.24 | 0.064% | -23.1% |  | 4.68 | 0.241% | -87.4% |  | 4.78 | 0.246% | -89.2% |
| Memorandum Items (2021-2040): |  |  |  |  |  |  |  |  |  |  |  |  |  |  |
| - Real GDP/capita/year (USD) | 1,939 |  |  |  |  |  |  |  |  |  |  |  |  |  |
| - NPV GDP (bn USD) | 68,198 |  |  |  |  |  |  |  |  |  |  |  |  |  |
| Notes: Own calculations; *We use "DB" to refer to "Total Disease Burden impact" on any given economic, epidemiological, and demographic indicator. | | | | | | | | | | | | | | |

| **Table G. 2021-2040 NPV of GDP - sensitivity impacts - pan-TB treatment and 9% case detection scenarios** | | | | | | | | | | | | | | |
| --- | --- | --- | --- | --- | --- | --- | --- | --- | --- | --- | --- | --- | --- | --- |
|  | Disease Burden (DB*) | |  | Scenario: pan-TB treatment | | |  | Scenario: 90% case detection | | |  | Scenario: combined | | |
| Indicators |  |  |  |  |  |  |  |  |  |  |  |  |  |  |
| ECONOMIC |  |  |  |  |  |  |  |  |  |  |  |  |  |  |
| - ΔNPV of GDP (2021-2040) (bn USD) | bn USD | % change |  | bn USD | % change | % of DB* |  | bn USD | % change | % of DB* |  | bn USD | % change | % of DB* |
| Baseline calibration 1 | -146.42 | -0.215% |  | 35.34 | 0.052% | -24.1% |  | 120.22 | 0.176% | -82.1% |  | 124.19 | 0.182% | -84.8% |
| Sensitivity calibration 2 | -115.93 | -0.170% |  | 27.77 | 0.041% | -24.0% |  | 94.31 | 0.138% | -81.3% |  | 97.41 | 0.143% | -84.0% |
| Sensitivity calibration 3 | -181.34 | -0.266% |  | 44.05 | 0.065% | -24.3% |  | 149.94 | 0.220% | -82.7% |  | 154.90 | 0.227% | -85.4% |
| Sensitivity calibration 4 | -143.12 | -0.210% |  | 35.34 | 0.052% | -24.7% |  | 108.66 | 0.159% | -75.9% |  | 114.60 | 0.168% | -80.1% |
| Sensitivity calibration 5 | -114.67 | -0.168% |  | 28.64 | 0.042% | -25.0% |  | 84.35 | 0.124% | -73.6% |  | 89.58 | 0.131% | -78.1% |
| Sensitivity calibration 6 | -176.03 | -0.258% |  | 36.30 | 0.053% | -20.6% |  | 135.72 | 0.199% | -77.1% |  | 142.77 | 0.209% | -81.1% |
| Sensitivity calibration 7 | -150.91 | -0.221% |  | 34.42 | 0.050% | -22.8% |  | 130.53 | 0.191% | -86.5% |  | 133.15 | 0.195% | -88.2% |
| Sensitivity calibration 8 | -120.42 | -0.177% |  | 27.25 | 0.040% | -22.6% |  | 103.12 | 0.151% | -85.6% |  | 105.19 | 0.154% | -87.4% |
| Sensitivity calibration 9 | -186.15 | -0.273% |  | 42.80 | 0.063% | -23.0% |  | 162.21 | 0.238% | -87.1% |  | 165.48 | 0.243% | -88.9% |
| Memorandum Items (2021-2040): |  |  |  |  |  |  |  |  |  |  |  |  |  |  |
| - Real GDP/capita/year (USD) | 1,939 |  |  |  |  |  |  |  |  |  |  |  |  |  |
| - NPV GDP (bn USD) | 68,198 |  |  |  |  |  |  |  |  |  |  |  |  |  |
| Notes: Own calculations; *We use "DB" to refer to "Total Disease Burden impact" on any given economic, epidemiological, and demographic indicator. | | | | | | | | | | | | | | |

References

1. WHO. 2024. Electronic Data. WHO TB Burden Estimates. World Health Organization: Geneva. URL: <https://www.who.int/teams/global-tuberculosis-programme/data#csv_files> (accessed 25 April 2024)
