# Supplementary material for: Estimating the health and macroeconomic burdens of tuberculosis in India, 2021–2040: A fully integrated modelling study
Source: PLoS Med. 2024 Dec 12;21(12):e1004491. doi: 10.1371/journal.pmed.1004491 (PMC11637336; doi:10.1371/journal.pmed.1004491)
Supplement: S5 Appendix — (DOCX) [file pmed.1004491.s006.docx]

## S5 Appendix. Sensitivity Analysis: TB patient Presenteeism

**Supplement to:**

Estimating the health and macroeconomic burdens of tuberculosis in India, 2021-2040: A fully-integrated modelling study

**Authors:**

Marcus R. Keogh-Brown, Tom Sumner, Sedona Sweeney, Anna Vassall, Henning Tarp Jensen,

**Correspondence:**

Marcus Keogh-Brown

Faculty of Public Health and Policy

London School of Hygiene & Tropical Medicine

London

UK

Email: marcus.keogh-brown@lshtm.ac.uk

It has been suggested that TB patients will generally be less productive at work during a period in the run-up to the start of their treatment, and that this productivity loss amounts on average to 48 pre-treatment workdays [1]. In this appendix, we present results where we re-simulate the disease burden and policy scenario results, presented in Tables 1 and 2 in the main body text. We use the same model, with the same set of fitted and calibrated parameter values, but with the important difference that every TB patient, who would have otherwise worked, will be less productive, and that their loss of productivity will be equivalent to a loss of 48 pre-treatment working-days. This additional morbidity-related productivity loss means that among DS and MDR patients, who are already assumed, on average, to lose 45 workdays during their treatment period, the need to take time off work, or to reduce their activity (and productivity) during the pre-treatment period, will increase their morbidity-related productivity loss by >100% (106.5% to be precise).

The re-simulated disease burden and policy scenario results, which follow from the change in morbidity parameter assumption, are presented in Tables A and B. We discuss the impact on our measurement of disease burdens in the first sub-section, and we subsequently discuss the impact of the policy scenario results in the following sub-section, before providing some final comments and conclusions

### Disease Burden impacts

The estimated aggregate disease burden impacts can be seen from columns 2-3 in Table A, while the breakdown across household income quintiles is presented in Table B.

The macroeconomic disease burden impacts, measured by 2021-2040 accumulated real GDP per capita, amount to respectively 4.79USD and 166.4bn USD. In comparison with the baseline results from Table 1, i.e. 4.21USD and 146.4bn USD, this means that the additional productivity loss amounts to an additional burden of 0.58USD per capita or 20.0bn USD, or increased macroeconomic burdens of 13.6%-13.7%.

If we zoom-in on the labour-related morbidity impact on NPV GDP, we can see that it amounted to 18.4bn USD without accounting for presenteeism (Table 1) and 38.8bn USD after accounting for presenteeism effects (Table A). This demonstrates that the drop in labour income resulting from TB patient pre-treatment presenteeism accounts fully for the overall decline in NPVGDP.

We can also confirm that the importance of mortality-related labour supply losses, for the overall NPVGDP disease burden of TB, has declined from approximately 77% (Table 1) to 68% (Table A), while the importance of morbidity-related labour supply- losses has increased from 12.5% (Table 1) to 23.3% (Table A), there demonstrating the potential importance of accounting for presenteeism when measuring the macroeconomic disease burden of TB.

If we look at Indian households, we can see that the burden on Indian household incomes has increased from US$116.8bn (Table 1) to 132.9bn USD (Table A), but the breakdown across household income quintiles (Tables 2 and B) continues to be progressive in absolute terms (highest absolute burden falling on the highest income quintile Q5; US$78.4bn (Table B) vs. US$69.0bn (Table 2)) and regressive in relative terms (highest relative burden falling on the lowest income quintile Q1: 0.51% (Table B) vs. 0.45% (Table 2)). For non-economic indicators, there has been no change in the health and demographic disease burden, and these indicators have therefore not changed as part of the sensitivity analysis. It follows that the non-economic TB burden remains regressive, in both absolute and relative terms.

In general, income burdens increase relatively equally across households by between 12.9%-13.6% (comparing Tables 2 and B), and this means that the conclusions, from the main body text, remain valid. Hence, the findings that absolute economic burdens are progressive, and that TB places higher health and relative income burdens on low-income households but larger total costs on high income households are robust.

### Policy Scenario Estimates: Pan-TB treatment, 90% detection rate, and combined scenario impacts

The macroeconomic impacts of the three policy scenarios, including pan-TB treatment, 90% case detection, and combined policy scenarios, are all sensitive to the increased morbidity-related productivity losses from presenteeism. Due to increased clinical health and economic disease burdens, it is no surprise that all policy scenarios have improved macroeconomic impacts. In terms of NPVGDP, roll-out of Pan-TB treatment would lead to a gain of US$40.3bn (Table A) compared to US$35.3bn without presenteeism accounted for (Table 1), achievement of the 90% case detection rate would lead to a gain of US$135.1bn (Table A) compared to US$120.2bn (Table 1), and roll-out of both scenarios combined would lead to a gain of US$139.5bn (Table A) compared to US$124.2bn without presenteeism accounted for (Table 1). Presenteeism therefore raises macroeconomic gains from our policy scenarios by 12.3%-14.2%. This is in line with the sensitivity of the underlying economic disease burden which was discussed in the previous section, and where we saw that macroeconomic and household income burdens grew by a similar order of magnitude, and our results confirm that the impact of our policy scenarios are sensitive to the morbidity-related productivity losses and that this translates into proportionally larger impacts of the proposed policy interventions across the board.

### Sensitivity Analysis summary

In summary, we have demonstrated the economic disease burdens are sensitive to accounting for pre-treatment presenteeism of TB patients. Overall, macroeconomic and household income burdens are likely to rise by 13-15% but, while the distribution of burdens among household income quintiles is likely to remain fairly unaffected, non-economic clinical, health, and demographic burdens are not affected.

At the same time, due to the increased economic burdens, there is a greater potential for our policy scenarios to improve economic outcomes, and our results confirm that the potential macroeconomic and household income impacts of all policy scenarios increase by roughly 12%-14%, in line with the increases in underlying economic burdens.

Overall, our sensitivity analyses show that productivity losses from pre-treatment presenteeism among TB patients has a potentially significant impact on economic disease burdens, and that this may, in turn, increase the economic benefit of policy interventions to control the burden. Hence, this should be taken into account when analysing the impact of policy interventions.

| **Table A. 2021-2040 economic, clinical, epidemiological and demographic impacts - pan-TB treatment and 90% case detection scenarios - Sensitivity: TB patient Presenteeism** | | | | | | | | | | | | | | |
| --- | --- | --- | --- | --- | --- | --- | --- | --- | --- | --- | --- | --- | --- | --- |
|  | Disease Burden (DB*) | |  | Scenario: pan-TB treatment | | |  | Scenario: 90% case detection | | |  | Scenario: combined | | |
| Indicators |  |  |  |  |  |  |  |  |  |  |  |  |  |  |
| ECONOMIC |  |  |  |  |  |  |  |  |  |  |  |  |  |  |
| GDP PER CAPITA (USD) | USD | % change |  | USD | % change | % of DB* |  | USD | % change | % of DB* |  | USD | % change | % of DB* |
| - ∆Real GDP/capita/year | -4.79 | -0.247% |  | 1.17 | 0.060% | -24.4% |  | 3.90 | 0.201% | -81.5% |  | 4.02 | 0.208% | -84.1% |
| NPV of GDP (2021-2040) (bn USD) | bn USD | % change |  | bn USD | % change | % of DB* |  | bn USD | % change | % of DB* |  | bn USD | % change | % of DB* |
| - ∆NPV GDP | -166.4 | -0.244% |  | 40.3 | 0.059% | -24.2% |  | 135.1 | 0.198% | -81.2% |  | 139.5 | 0.205% | -83.8% |
| - ∆NPV GDP (Treatment Costs) | -15.7 | -0.023% |  | 3.2 | 0.005% | -20.6% |  | 11.2 | 0.016% | -71.0% |  | 11.5 | 0.017% | -73.4% |
| - ∆NPV GDP (∆Labour supply - Morbidity) | -38.8 | -0.057% |  | 9.3 | 0.014% | -24.0% |  | 28.3 | 0.042% | -72.9% |  | 29.1 | 0.043% | -74.9% |
| - ∆NPV GDP (∆Labour supply - Mortality) | -113.3 | -0.166% |  | 27.6 | 0.040% | -24.3% |  | 95.6 | 0.140% | -84.3% |  | 98.8 | 0.145% | -87.2% |
| NPV of hhld income and consumption (2021-2040) (bn USD) | bn USD | % change |  | bn USD | % change | % of DB* |  | bn USD | % change | % of DB* |  | bn USD | % change | % of DB* |
| - ∆NPV Household income | -132.9 | -0.195% |  | 32.1 | 0.056% | -24.2% |  | 107.9 | 0.187% | -81.2% |  | 111.4 | 0.193% | -83.8% |
| - ∆NPV Labour income | -76.0 | -0.111% |  | 18.2 | 0.048% | -24.0% |  | 61.5 | 0.163% | -81.0% |  | 63.6 | 0.168% | -83.6% |
| - ∆NPV Capital income | -56.9 | -0.083% |  | 13.9 | 0.070% | -24.5% |  | 46.3 | 0.234% | -81.5% |  | 47.8 | 0.241% | -84.1% |
| - ∆NPV Household consumption | -116.2 | -0.170% |  | 28.2 | 0.065% | -24.2% |  | 94.4 | 0.218% | -81.2% |  | 97.4 | 0.225% | -83.8% |
| Labour market (1000s person-years) | 1000s | % change |  | 1000s | % change | % of DB* |  | 1000s | % change | % of DB* |  | 1000s | % change | % of DB* |
| - ∆Unskilled labour (#person-years) | -32,911.5 | -0.360% |  | 8,283 | 0.091% | -25.2% |  | 27,266 | 0.298% | -82.8% |  | 28,117 | 0.308% | -85.4% |
| - ∆Skilled labour (#person-years) | -5,163.7 | -0.256% |  | 1,283 | 0.063% | -24.9% |  | 4,255 | 0.211% | -82.4% |  | 4,390 | 0.217% | -85.0% |
| **CLINICAL, EPIDEMIOLOGICAL & DEMOGRAPHIC** |  |  |  |  |  |  |  |  |  |  |  |  |  |  |
| Demographic outcomes | 1000s | % of total |  | 1000s | % of total | % of DB* |  | 1000s | % of total | % of DB* |  | 1000s | % of total | % of DB* |
| - ∆Population (1000s prs-years) | -80,029.5 | -0.27% |  | 19,922 | 0.07% | -24.9% |  | 68,121 | 0.23% | -85.1% |  | 70,382 | 0.23% | -87.9% |
| - Excess deaths (1000s persons) | 7,042.4 | 3.58% |  | -2,187 | -1.11% | -31.1% |  | -6,275 | -3.19% | -89.1% |  | -6,426 | -3.27% | -91.2% |
| Clinical Outcomes (1000 persons) | 1000s | % change |  | 1000s | % change | % of DB* |  | 1000s | % change | % of DB* |  | 1000s | % change | % of DB* |
| - ∆TB incident cases | 62,424 | - |  | -16,214 | -26.0% | -26.0% |  | -47,294 | -75.8% | -75.8% |  | -48,475 | -77.7% | -77.7% |
| - ∆TB case fatalities | 8,102 | - |  | -2,518 | -31.1% | -31.1% |  | -7,209 | -89.0% | -89.0% |  | -7,384 | -91.1% | -91.1% |
| Cumulative population by compartment (mm pers-yrs†) | mm | % change |  | mm | % change | % of DB* |  | mm | % change | % of DB* |  | mm | % change | % of DB* |
| - ∆Susceptible population | 18,239.3 | - |  | 555.1 | 3.0% | 3.0% |  | 1,955.4 | 10.7% | 10.7% |  | 2,022.1 | 11.1% | 11.1% |
| - ∆Latently infected population | 11,703.2 | - |  | -497.8 | -4.3% | -4.3% |  | -1,787.9 | -15.3% | -15.3% |  | -1,848.1 | -15.8% | -15.8% |
| - ∆Infectious population ‡ | 95.8 | - |  | -29.8 | -31.1% | -31.1% |  | -85.2 | -89.0% | -89.0% |  | -87.3 | -91.1% | -91.1% |
| - ∆Treatment population $ | 25.4 | - |  | -7.6 | -30.1% | -30.1% |  | -14.3 | -56.1% | -56.1% |  | -16.3 | -64.2% | -64.2% |
| Risk factors (endogenous) | %-points | % change |  | %-points | % change | % of DB* |  | %-points | % change | % of DB* |  | %-points | % change | % of DB* |
| - ∆low BMI prevalence (avg) | 20.45% | - |  | -0.36% | -1.8% | -1.8% |  | -1.18% | -5.8% | -5.8% |  | -1.22% | -6.0% | -6.0% |
| Memorandum Items (2021-2040): |  |  |  |  |  |  |  |  |  |  |  |  |  |  |
| - Real GDP/capita/year (USD) | 1,939 |  |  |  |  |  |  |  |  |  |  |  |  |  |
| - NPV GDP (bn USD) | 68,198 |  |  |  |  |  |  |  |  |  |  |  |  |  |
| Notes: Own calculations; *We use "DB" to refer to "Total Disease Burden impact" on any given economic, epidemiological, and demographic indicator; †We use "mm prs-yrs" to refer to millions of cumulative person-years over our 2021-40 time horizon; ‡ The infectious population consists of both newly infectious and previously unsuccessfully treated persons; $ The treatment population consists of both persons who are newly treated and re-treated after prior unsuccessful treatment. | | | | | | | | | | | | | | |

| **Table B. 2021-2040 cumulative household impacts - TB Disease Burden (bn USD; 2021 prices) – Sensitivity: TB patient presenteeism** | | | | | | | | | | | | | | |
| --- | --- | --- | --- | --- | --- | --- | --- | --- | --- | --- | --- | --- | --- | --- |
|  | Households | | | | | | | | | | | | | |
|  | Income Q1 | |  | Income Q2 | |  | Income Q3 | |  | Income Q4 | |  | Income Q5 | |
|  |  |  |  |  |  |  |  |  |  |  |  |  |  |  |
| ECONOMIC DISEASE BURDEN | bn USD | % of base |  | bn USD | % of base | | bn USD | % of base | | bn USD | % of base | | bn USD | % of base |
| - ∆NPV Household income | 10.5 | 0.51% |  | 11.4 | 0.33% |  | 13.1 | 0.25% |  | 19.5 | 0.20% |  | 78.4 | 0.21% |
| - ∆NPV Labour income | 10.2 | 0.52% |  | 10.4 | 0.34% |  | 10.7 | 0.24% |  | 13.3 | 0.18% |  | 31.5 | 0.15% |
| - ∆NPV Capital income | 0.3 | 0.29% |  | 1.0 | 0.29% |  | 2.4 | 0.29% |  | 6.2 | 0.29% |  | 46.9 | 0.29% |
| - ∆NPV Household consumption | 10.2 | 0.54% |  | 11.3 | 0.37% |  | 13.1 | 0.28% |  | 19.3 | 0.23% |  | 62.3 | 0.24% |
| LABOUR MARKET | 1000s | % of base | |  | 1000s | | % of base |  | | 1000s | % of base | |  | 1000s |
| - ∆Unskilled labour (#person-years) | 9,760 | 0.64% |  | 8,537 | 0.44% |  | 6,792 | 0.33% |  | 4,795 | 0.24% |  | 3,027 | 0.19% |
| - ∆Skilled labour (#person-years) | 464 | 0.64% |  | 715 | 0.44% |  | 822 | 0.33% |  | 1,117 | 0.24% |  | 2,046 | 0.19% |
| DEMOGRAPHY |  |  |  |  |  |  |  |  |  |  |  |  |  |  |
| - ∆Population (#person-years) | 22,040 | 0.47% |  | 19,377 | 0.34% |  | 15,911 | 0.26% |  | 12,286 | 0.19% |  | 10,416 | 0.15% |
| - Excess deaths (#persons) | -1,857 | -5.02% |  | -1,752 | -5.59% |  | -1,428 | -4.06% |  | -1,095 | -2.69% |  | -910 | -1.74% |
| TB CLINICAL OUTCOMES |  |  |  |  |  |  |  |  |  |  |  |  |  |  |
| - TB incident cases (#persons) | -16,591 | 27% |  | -15,411 | 25% |  | -12,586 | 20% |  | -9,706 | 16% |  | -8,130 | 13% |
| - TB case fatalities (#persons) | -2,154 | 27% |  | -2,001 | 25% |  | -1,633 | 20% |  | -1,259 | 16% |  | -1,055 | 13% |
| Note: Own calculations |  |  |  |  |  |  |  |  |  |  |  |  |  |  |

References

1. Sinha P, Carwile M, Bhargava A, Cintron C, Acuna-Villaorduna C, Lakshminarayan S, et al. *How much do Indians pay for tuberculosis treatment? A cost analysis*. Public Health Action, 2020. 10(3):110-17. DOI: <https://doi.org/10.5588/pha.20.0017>.
