# Supplementary material for: Estimating the health and macroeconomic burdens of tuberculosis in India, 2021–2040: A fully integrated modelling study
Source: PLoS Med. 2024 Dec 12;21(12):e1004491. doi: 10.1371/journal.pmed.1004491 (PMC11637336; doi:10.1371/journal.pmed.1004491)
Supplement: S6 Appendix — (DOCX) [file pmed.1004491.s007.docx]

## S6 Appendix. Existing Economic Methodologies

**Supplement to:**

Estimating the health and macroeconomic burdens of tuberculosis in India, 2021-2040: A fully-integrated modelling study

**Authors:**

Marcus R. Keogh-Brown, Tom Sumner, Sedona Sweeney, Anna Vassall, Henning Tarp Jensen,

**Correspondence:**

Marcus Keogh-Brown

Faculty of Public Health and Policy

London School of Hygiene & Tropical Medicine

London

UK

Email: marcus.keogh-brown@lshtm.ac.uk

Two of the main methodologies which have been used to estimate the economic burden of TB are the Value of Statistical Life (VSL) and Cost of Illness approaches. The VSL approach weights the value that individuals attach to their lives by the number of life years lost due to a disease to yield a single monetary estimate of the disease burden. This single estimate is multiplied by the change in life expectancy or time over which mortality was averted. The first global VSL estimates of the full income losses due to TB was estimated for 120 countries from 2020 to 2050 to be US$17.5 trillion [1]. Estimates for India obtained by this method estimated that from 2020 to 2050, India would suffer 53,789,255 incident cases and 8,763,539 TB-related deaths at a cost of US$6.63 trillion in 2020 prices. Whilst the underlying epidemiological estimates differ from our TB model indicating just 86% of the incidence and 92% of the deaths estimated by our model would occur over a 30 year period, the VSL economic burden is more than 45 times larger which highlights the significant difference between our pecuniary estimation approach and valuations which assign willingness to pay estimates to both employed and dependent population members. The COI approach is based upon individual level costs and involves summing direct and indirect costs of a disease over a fixed period. The COI approach has been used in the context of TB to estimate costs from the patient [2-4], provider [5] and societal perspective [6-8]. By this method total spending including government, out of pocket spending and development assistance for TB in LMICs in 2017, has been estimated at $10.9 billion, $1.9billion in India [9]. Total Tuberculosis spending per incident case in India for the COI estimate is $644 which compares to our macroeconomic estimate of $2345. This agrees with the WHO and others who have highlighted the inapplicability of the COI approach for macroeconomic analysis since their limited focus on health sector spending and lost labour productivity only, provides a partial picture of the true macroeconomic impact of disease and excludes depleted capital accumulation, investment in human capital and demographic change to diminished economic growth [10]. Furthermore, the inability of the COI approach to capture general equilibrium adjustment mechanisms at the macroeconomic level or individual behavioural changes and policy has also been highlighted [11] For these reasons a general equilibrium approach is recommended for macroeconomic valuations [10, 12].

The strengths of using Computable General Equilibrium (CGE) models in health applications includes that they are able to simultaneously capture the impact of changes in healthcare expenditures, health-related investments, and health-related changes in availability or productivity of labour across all sectors of the economy. When linked to demographic models, as in our case, CGE models can additionally distinguish between impacts, such as health and care effects on those outside working age and the long-term effects of population change on future labour supply. Correctly identifying these labour supply impacts for a disease such as TB is important for accurate cost estimation. Previously CGE models have been applied which seek to account for the impact of TB in their analyses [13, 14]. However, such studies are rare and focus on the economic implications of anti-microbial resistance (AMR) for several diseases, including HIV, TB, and Malaria. They lack application of detailed epidemiological modelling of disease and do not isolate outcomes for individual countries. Therefore, to our knowledge, the current study is the first macroeconomic CGE modelling study of TB in India which captures treatment impacts.

References

1. Silva S, Arinaminpathy N, Atun R, Goosby E, Reid M. *Economic impact of tuberculosis mortality in 120 countries and the cost of not achieving the Sustainable Development Goals tuberculosis targets: a full-income analysis.* Lancet Glob Health, 2021. **9**(10): p. e1372-e1379. DOI: <https://doi.org/10.1016/S2214-109X(21)00299-0>.

2. Kik SV, Olthof SPJ, de Vries JTN, Menzies D, Kincler N, Loenhout-Rooyakkers J, et al. *Direct and indirect costs of tuberculosis among immigrant patients in the Netherlands.* BMC Public Health, 2009. **9**(1):283. DOI: <https://doi.org/10.1186/1471-2458-9-283>.

3. Laokri S, Dramaix-Wilmet M, Kassa F, Anagonou S, Dujardin B. *Assessing the economic burden of illness for tuberculosis patients in Benin: determinants and consequences of catastrophic health expenditures and inequities.* Trop Med Int Health, 2014. **19**(10):1249-1258. DOI: <https://doi.org/10.1111/tmi.12365>.

4. Mauch V, Woods N, Kirubi B, Kipruto H, Sitienei J, Klinkenberg E. *Assessing access barriers to tuberculosis care with the Tool to Estimate Patients' Costs: pilot results from two districts in Kenya.* BMC Public Health, 2011. **11**(1):43. DOI: <https://doi.org/10.1186/1471-2458-11-43>.

5. Bay V, Tabarsi P, Rezapour A, Marzban S, Zareid E. *Cost of Tuberculosis Treatment: Evidence from Iran's Health System.* Osong Public Health Res Perspect, 2017. **8**(5): p. 351-357. DOI: https://doi.org/[10.24171/j.phrp.2017.8.5.09](https://doi.org/10.24171/j.phrp.2017.8.5.09).

6. Hasoumi M, Nasehi M, Khakian M, Mohseni M, Ziaiifar H, Keykale MS. *Cost of illness of tuberculosis in tehran in the year 2011.* Mater Sociomed, 2014. **26**(5): p. 339-42.

7. John, K.R., et al., *Costs incurred by patients with pulmonary tuberculosis in rural India.* Int J Tuberc Lung Dis, 2009. **13**(10):1281-1287. DOI: <https://doi.org/10.5455/msm.2014.26.339-342>.

8. Zannetos S, Zachariadou T, Adamidi T, Georgiou A. *The economic burden of tuberculosis in Cyprus. A probabilistic cost of illness study.* Epidemiol Biostat Public Health, 2018. **15**(2). DOI: <https://doi.org/10.2427/12780>.

9. Su Y, Baena IG, Harle AC, Crosby SW, Micah AE, Siroka A, et al. *Tracking total spending on tuberculosis by source and function in 135 low-income and middle-income countries, 2000-17: a financial modelling study.* Lancet Infect Dis, 2020. **20**(8):929-942. DOI: <https://doi.org/10.1016/S1473-3099(20)30124-9>.

10. WHO. *WHO guide to identifying the economic consequences of disease and injury.* Geneva: World Health Organization, 2009. URL: <https://www.who.int/publications/i/item/9789241598293>. (accessed 28. October 2024)

11. Bloom DE, Kuhn M, Prettner K. *Modern infectious diseases: macroeconomic impacts and policy responses.* J Econ Lit, 2022. **60**(1):85-131. DOI: <https://doi.org/10.1257/jel.20201642>.

12. Kotsopoulos, N. Connolly, M.P., *Is the gap between micro- and macroeconomic assessments in health care well understood? The case of vaccination and potential remedies.* J Mark Access Health Policy, 2014 Apr 10; **2**. DOI: <https://doi.org/10.3402/jmahp.v2.23897>.

13. Ahmed SA, Baris E, Go DS, Lofgren H, Osorio-Rodarte I, Thierfelder K. *Assessing the global poverty effects of antimicrobial resistance.* World Development 111:148-160. DOI: <https://doi.org/10.1016/j.worlddev.2018.06.022>.

14. Taylor J, Hafner M, Yerushalmi E, Smith RD, Bellasio J, Vardavas R, et al. *Estimating the economic costs of antimicrobial resistance: model and results.* Rand Europe Research Repport, 2014. URL: <https://www.rand.org/pubs/research_reports/RR911.html>. (accessed 28. October 2024)
